# Coupling oxidative de-stabilization with immune activation overcomes radioresistance in hepatocellular carcinoma

**DOI:** 10.1186/s12943-026-02729-z

**Published:** 2026-07-30

**Authors:** Jiahao Liu, Ze Mi, Hongpei Tan, Junhong Duan, Feilong Huang, Shahan Guo, Yuting Li, Jianmin Wu, Min Yang, Yunjie Liao, Xiaoqian Ma, Qi Liang, Weiwei Zhang, Xiaofei Fan, Pengfei Rong

**Affiliations:** 1 Department of Radiology, Central South University, The Third Xiangya HospitalNo. 138, Tongzipo Road, Changsha, 410013 Hunan China; 2 Department of Diagnostic Radiology, Yong Loo Lin School of Medicine, National University of Singapore, Singapore, 119074 Singapore; 3 Postdoctoral Station of Medical Aspects of Specific Environments, the Third Xiangya Hospital, Central South University, Changsha, China; 4 Shandong Medical College, No. 5460, Second Ring South Road, Jinan, 250002 Shandong China; 5 Transplantation Center, The Third Xiangya Hospital, Central South University, No. 138, Tongzipo Road, Changsha, 410013 Hunan China; 6 Department of Clinical Laboratory, Shandong Cancer Hospital and Institute, Shandong First Medical University, and Shandong Academy of Medical Sciences, Jinan, Shandong, 250117 China; 7 Furong Laboratory, Changsha, 410008 Hunan China

**Keywords:** Radioresistance, Ferroptosis, Immunogenic cell death, HCC

## Abstract

**Background:**

Radioresistance in hepatocellular carcinoma (HCC) is a coordinated adaptive state rather than failure of a single pathway, sustained by reinforced redox buffering and suppressed immune activation. Strategies that intensify radiation damage without disrupting this state have shown limited efficacy. We sought to determine whether radiosensitization can be achieved by destabilizing tumor oxidative homeostasis while restoring immune competence.

**Methods:**

A BSA-templated Fe/Mn nanoassembly incorporating the thiol-reactive agent RRX-001 and folate targeting (BFMR-FA) was developed as a multifunctional and MRI-visible platform. Its biological effects were evaluated across radioresistant cellular systems, organoids, and orthotopic tumor models.

**Results:**

BFMR-FA shifts the redox equilibrium of tumor cells beyond a recoverable threshold. By depleting glutathione and suppressing the SLC7A11–GPX4 axis, it converts radiation-induced oxidative stress from a transient signal into sustained lipid peroxidation, resulting in convergent ferroptotic and apoptotic cell death. This transition is accompanied by the emergence of immunogenic stress signals and activation of the STING–TBK1–IRF3 pathway, enabling dendritic cell maturation and expansion of cytotoxic T-cell responses. In vivo, these effects translate into near-complete suppression of radioresistant tumor growth with systemic tolerance. The Fe/Mn composition enables direct visualization of intratumoral delivery by T1-weighted MRI.

**Conclusion:**

These findings support a model in which radiosensitization is achieved not by amplifying tumor damage alone, but by collapsing the adaptive redox state that constrains it. The resulting oxidative destabilization coupled with immune activation defines an oxidative homeostasis collapse–driven radio-immuno synergy, providing a generalizable framework for overcoming therapeutic resistance in solid tumors.

**Supplementary Information:**

The online version contains supplementary material available at https://doi.org/10.1186/s12943-026-02729-z.

## Background

Radiotherapy is a cornerstone in the treatment of solid tumors, including hepatocellular carcinoma (HCC), where it plays an important role in disease management [1, 2]. Despite significant advances in dose delivery, imaging guidance, and treatment precision, durable therapeutic responses remain limited [3]. Radioresistance remains a considerable barrier, contributing to local treatment failure and disease recurrence [4, 5]. Importantly, this resistance is not simply the result of insufficient DNA damage; instead, it reflects a complex adaptive state of tumor cells that enables survival under genotoxic stress while actively suppressing antitumor immunity [6, 7].

A hallmark of radioresistant tumors is their strengthened oxidative homeostasis [8, 9]. Ionizing radiation exerts much of its cytotoxic effect through the generation of reactive oxygen species (ROS), which induce DNA damage and lipid peroxidation [10–12]. However, most tumor cells effectively neutralize this insult by upregulating antioxidant defenses, including enhanced glutathione biosynthesis, GPX4-mediated detoxification of lipid peroxides, and metabolic rewiring that buffers redox stress [13, 14]. Consequently, radiation-induced ROS are neutralized before reaching lethal thresholds, thereby reducing therapeutic efficacy. Efforts to overcome this barrier have focused primarily on increasing ROS production or inhibiting individual antioxidant enzymes [15–17]. Still, these approaches have yielded limited clinical success due to pathway redundancy and rapid adaptive compensation.

In parallel, radiotherapy often fails to elicit robust antitumor immunity [18, 19]. Although radiation can induce immunogenic cell death (ICD), this response is frequently attenuated in radioresistant tumors characterized by immune exclusion, defective antigen presentation, and suppression of innate immune sensing pathways, such as cGAS–STING [20–22]. Consequently, radiotherapy typically functions as a local cytotoxic intervention rather than a catalyst for durable, systemic immune-mediated tumor control.

Together, these observations underscore a fundamental limitation of current radiosensitization strategies. Radioresistance is not governed by a single pathway but by a systems-level adaptive state that integrates redox buffering, lipid peroxide detoxification, flexible DNA repair, and immune suppression [23–25]. Incremental disruption of individual nodes within this network is therefore unlikely to produce sustained therapeutic benefit. Instead, effective radiosensitization may require coordinated destabilization of tumor oxidative homeostasis coupled with reactivation of antitumor immunity.

Ferroptosis, an iron-dependent form of regulated cell death driven by unchecked lipid peroxidation, is a promising vulnerability in therapy-resistant cancers [26, 27]. Unlike apoptosis, ferroptosis directly exploits redox imbalance to generate lipid-derived danger signals that can amplify ICD and innate immune activation [7, 28]. However, inducing ferroptosis alone is often insufficient in *vivo* due to residual antioxidant capacity and the persistence of an immunosuppressive tumor microenvironment [29, 30]. We hypothesized that synchronizing ferroptosis induction with immune reprogramming during radiotherapy could overcome entrenched resistance mechanisms.

To test this concept, we developed a multifunctional nanoplatform that integrates iron-driven lipid peroxidation, a clinically validated redox-modulating agent, and tumor-targeting capabilities. This system is engineered to collapse tumor oxidative homeostasis upon radiation exposure, promote ferroptosis–apoptosis convergence, and initiate immune activation. The incorporation of MRI traceability further enables real-time assessment of intratumoral delivery and therapeutic engagement, addressing a key translational challenge in nanomedicine [31].

Using hepatocellular carcinoma as a clinically relevant model of radioresistance, we demonstrate that this approach establishes an oxidative homeostasis collapse–driven radio-immuno synergy (OHC-RIS). This framework mechanistically links redox destabilization, immunogenic cell death, and STING-dependent immune activation into a unified therapeutic paradigm, shifting radiotherapy from a purely local cytotoxic modality into a systemic, immune-amplified intervention.

## Materials and methods

### Cell culture

Bovine serum albumin (BSA), ferrous chloride tetrahydrate (FeCl₂·4H₂O), manganese(II) chloride tetrahydrate (MnCl₂·4H₂O), sodium hydroxide, and analytical-grade solvents were purchased from Sinopharm Chemical Reagent Co., Shanghai, China. RRX-001, Ferrostatin-1, Liproxstatin-1, Z-VAD-FMK, N-acetyl-L-cysteine, Bafilomycin A1, and 3-methyladenine were purchased from Selleck Chemicals, Houston, USA, and only reagents with experimentally verified catalog numbers were used. DMEM, RPMI-1640, fetal bovine serum, and penicillin–streptomycin were purchased from Thermo Fisher Scientific (Waltham, USA). ELISA kits for IFN-γ, TNF-α, IL-12, IL-10, Granzyme B, and TGF-β were purchased from Aifang BioTech (Changsha, China). Tumor tissues were dissociated using the BD Tumor Dissociation Kit (BD Biosciences, San Jose, USA). HuH-7, Hepa1-6, AML12, and HEK293T cell lines were obtained from Central South University, Changsha, China. Radioresistant RR-Huh7 and RR-Hepa1-6 cells were generated by repeated fractionated irradiation.

### Radiation-resistant HuH7 and Hepa1-6 cell line establishment

Radiation-resistant HuH7 and Hepa1-6 cell lines were generated through repeated fractionated X-ray irradiation. Briefly, exponentially growing HuH7 and Hepa1-6 cells were seeded into 25-cm^2^ culture flasks at 5 × 10^4^ cells per flask. After adherence and reaching 60–70% confluence, cells were irradiated with 4 Gy of X-rays using a small-animal irradiator. During irradiation, culture flasks were completely covered with culture medium to ensure sufficient medium depth above the cell monolayer, thereby minimizing potential surface dose variation caused by the air–medium interface or the polystyrene flask bottom. Fresh medium was replaced every 2 days. When confluence exceeded 85%, cells were trypsinized and passaged; 24 h after passaging, an additional 4 Gy of irradiation was administered. This fractionated irradiation cycle was repeated until a cumulative dose of 60 Gy was achieved. Cells were then maintained for 2–3 additional passages without irradiation to stabilize the resistant phenotype, yielding the radiation-resistant HuH7 and Hepa1-6 sublines. Parental HuH7 and Hepa1-6 cells were cultured in parallel without irradiation as controls.

### Animal studies

Six-week-old BALB/c nude mice and C57BL/6 mice were obtained from Hunan SJA Laboratory Animal Co. (Changsha, China) and maintained under specific pathogen-free (SPF) conditions with controlled temperature (20–26 °C), humidity (40–60%), and a 12 h light–dark cycle, with free access to food and water. For the subcutaneous tumor model, radiation-resistant RR-HuH7 cells were centrifuged at 300 × g for 5 min, washed with PBS, and resuspended at 1 × 10⁷ cells/mL. A total of 100 μL of the cell suspension was injected subcutaneously into the right axillary region of BALB/c nude mice using a 27-gauge needle. Tumor volume and body weight were monitored every two days, and tumor size was calculated using the formula (L × W2)/2. Mice were randomly assigned to treatment groups when tumors reached approximately 80–120 mm3.

For the orthotopic hepatocellular carcinoma (HCC) model, RR-Hepa1-6 cells in logarithmic growth were washed with PBS and resuspended in serum-free DMEM at a concentration of 2 × 10⁷ cells/mL. C57BL/6 mice were anesthetized with isoflurane, and a small abdominal incision (~ 1 cm) was made to expose the left liver lobe. A 20–30 μL cell suspension (~ 5 × 105 cells) was slowly injected into the hepatic parenchyma using a microsyringe. The injection site was gently compressed to prevent leakage, followed by sequential closure of the peritoneum and skin. During surgical procedures, animals were maintained under anesthesia and closely monitored during recovery to minimize distress. Humane endpoints were predefined, including tumor diameter exceeding 15 mm, body weight loss exceeding 20%, or signs of severe distress such as impaired mobility or persistent lethargy. Animals reaching these criteria were euthanized immediately according to institutional animal care guidelines.

All animal experiments were approved by the Animal Care and Use Committee of Central South University (Approval No. APU-2025–0479) and were conducted and reported in accordance with the ARRIVE guidelines.

### Synthesis of BFMR-FA nanoparticles

BFMR-FA nanoparticles were synthesized using a BSA-templated coordination strategy. Briefly, 100 mg of bovine serum albumin (BSA) was dissolved in 10 mL serum-free DMEM under vortex mixing and mild ultrasonication until complete dissolution. RRX-001 was then added to the solution to reach a final concentration of 10 mM. Subsequently, 40 μL of 1 M FeCl₂ solution was introduced, and the pH of the mixture was adjusted to 9.0 using NaOH. The reaction mixture was incubated for 4 h to allow coordination between Fe2⁺ ions and the BSA template. Afterward, 40 μL of 1 M MnCl₂ solution was added, and the mixture was further incubated at 37 °C in the dark for 24 h to enable co-incorporation of Mn ions into the nanostructure. The resulting nanoparticles were collected by centrifugation at 16,000 rpm for 30 min, and the supernatant was discarded. The precipitate was washed twice with ultrapure water to remove unbound components and then redispersed in ultrapure water, yielding RRX-001@BSA@FeMn nanoparticles (BFMR). For surface functionalization, 40 μL folic acid solution was added to the nanoparticle suspension and the mixture was stirred at 37 °C in the dark for 1 h. The final nanoparticles were harvested by centrifugation (16,000 rpm, 30 min) and washed twice with ultrapure water. The purified nanoparticles were resuspended in ultrapure water to obtain RRX-001@BSA@FeMn-FA (BFMR-FA).

### Characterization and stability assessment of BFMR-FA nanoparticles

The hydrodynamic diameter and polydispersity index (PDI) of BFM and BFMR-FA nanoparticles were measured by dynamic light scattering (DLS) using a Malvern Zetasizer Nano ZS. The zeta potential was determined by electrophoretic light scattering on the same instrument. All measurements were performed at 25 °C after appropriate dilution in ultrapure water. The morphology and size of BFMR-FA nanoparticles were examined by transmission electron microscopy (TEM, JEOL JEM-F200). For sample preparation, a drop of the diluted nanoparticle suspension was deposited onto carbon-coated copper grids and dried at room temperature. Elemental mapping and energy-dispersive X-ray spectroscopy (EDS) were conducted using a field-emission TEM equipped with an EDS detector to visualize the spatial distribution of Fe and Mn within the nanoparticles. X-ray photoelectron spectroscopy (XPS, Thermo Scientific K-Alpha) was employed to analyze the surface elemental composition and chemical states. Spectra were acquired using monochromatic Al Kα radiation, and all binding energies were calibrated to the C 1 s signal at 284.8 eV. Fourier transform infrared spectroscopy (FTIR, Thermo Scientific iN10) was used to identify characteristic functional groups of BFMR-FA. Spectra were collected in the range of 4000–600 cm⁻1 using the attenuated total reflection (ATR) mode. Ultraviolet–visible (UV–vis) absorption spectra were recorded using a Shimadzu UV-3600 spectrophotometer to evaluate the optical properties of BFMR-FA and confirm the folic acid modification.

Stability was assessed by dispersing BFMR-FA in deionized water, PBS containing 10% FBS, and DMEM supplemented with 10% FBS. Samples were collected at 0, 1, 2, 6, 12 24 and 48 h, and the hydrodynamic diameter and polydispersity index were measured by dynamic light scattering. Stability was assessed by temporal changes in particle size across the tested media.

### Toxicity assessment of BFMR-FA nanoparticles

To measure toxicity, the hemolytic activity of BFMR-FA was assessed using freshly collected EDTA-anticoagulated mouse blood. Whole blood was centrifuged at 1500 rpm for 10 min to isolate erythrocytes, which were then washed three times with PBS and resuspended to 2% (v/v). Equal volumes of the RBC suspension and BFMR-FA solutions (0–200 μg/mL) were mixed and incubated at 37 °C for 30 min. PBS and deionized water served as negative and positive controls, respectively. After incubation, samples were centrifuged at 1500 rpm for 10 min, and the hemoglobin released into the supernatant was quantified spectrophotometrically to calculate hemolysis percentages.

Serum Biochemistry Analysis and Hematology Analysis. At the end of treatment, whole blood was collected from mice via retro-orbital sampling. Serum was separated by centrifugation at 3000 rpm for 10 min and analyzed biochemically for albumin (Alb), alanine aminotransferase (ALT), aspartate aminotransferase (AST), γ-glutamyl transferase (γ-GT), blood urea nitrogen (BUN), creatinine (Cre), and creatine kinase-MB (CK-MB). In parallel, peripheral blood collected into EDTA-anticoagulated tubes was analyzed for complete blood counts (CBCs). Hematological parameters—including white blood cell count (WBC), granulocyte count (GRAN), granulocyte percentage (GRAN%), lymphocyte percentage (Ly%), red blood cell count (RBC), hemoglobin (HGB), mean corpuscular volume (MCV), mean corpuscular hemoglobin (MCH), mean corpuscular hemoglobin concentration (MCHC), platelet count (PLT), and hematocrit (HCT)—were quantified using an automated hematology analyzer according to standard protocols.

### Cell viability and apoptosis detection

Cell viability after BFMR-FA exposure and irradiation was measured using the Alamar Blue assay (Thermo Fisher Scientific). HuH-7 and RR-HuH-7C cells were cultured in RPMI-1640 or DMEM supplemented with 10% FBS and 1% penicillin–streptomycin under standard conditions (37 °C, 5% CO₂). Cells in the logarithmic growth phase were seeded into 96-well plates at 5 × 103 cells per well and incubated for 24 h to reach approximately 60% confluence. BFMR-FA was added at the indicated concentrations, and irradiation was performed 12 h later. After treatment, Alamar Blue reagent was added, and fluorescence was measured at 585 nm using an Infinite M200 PRO reader (TECAN).

Apoptosis was evaluated under the following treatment conditions: control, RT, RRX-001, RRX-001 + RT, BFMR-FA-FA, and BFMR-FA-FA + RT, using an Annexin V–FITC/PI staining protocol. After treatment, cells were harvested and washed twice with cold, tenfold-diluted PBS, then resuspended in a larger staining volume to ensure uniform dye exposure. Annexin V–FITC was added and incubated in the dark for 10 min, followed by PI for an additional 15 min. The stained suspensions were diluted with PBS, gently mixed, and immediately analyzed by flow cytometry using the standard FITC and PI channels. At least 10,000 events were collected for each sample, and apoptotic fractions were determined based on Annexin V and PI labeling patterns.

### Colony formation assay

Wild-type and oeGPX4 HuH-7 or Hepa1-6 cells were seeded in 6-well plates and allowed to adhere overnight. Cells were treated with BFMR-FA followed by X-ray irradiation, and then cultured for 10–14 days. Colonies were fixed with 4% paraformaldehyde for 15 min, stained with 0.1% crystal violet for 20 min, washed with PBS, and photographed. Colonies containing more than 50 cells were counted. Data were obtained from three independent experiments.

### FA competitive uptake assay

HuH-7, RR-HuH-7, Hepa1-6, RR-Hepa1-6, LO2, and AML-12 cells were seeded in confocal dishes and allowed to adhere overnight. For receptor-blocking experiments, cells were pretreated with excess free folic acid (FA, 1 mM) for 1 h, followed by incubation with Cy5.5-labeled BFMR or BFMR-FA for 4 h. Cells were then washed with PBS, fixed with 4% paraformaldehyde, counterstained with DAPI, and imaged by confocal microscopy under identical settings.

### Western blot and immunofluorescence staining

Cells were harvested after treatment and lysed in RIPA buffer containing protease inhibitors. Protein concentrations were determined using a BCA assay. Equal amounts of protein were mixed with SDS loading buffer, heated at 95 °C for 5–10 min, and separated by SDS–PAGE on 10–15% resolving gels. Proteins were transferred to PVDF membranes using a wet-transfer system at 300 mA for 1–2 h. Membranes were blocked with 5% skim milk or BSA for 1 h at room temperature and incubated overnight at 4 ℃ with the following primary antibodies (1:1000, Abcam): anti-CD86 (ab239075), anti-CD206 (ab64693), anti-4-HNE (ab46545), anti-SLC7A11 (ab175186), anti-GPX4 (ab125066), anti-γ-H2AX (ab81299), and anti-HIF-1α (ab51608). After three washes with TBST, membranes were incubated with HRP-conjugated secondary antibodies (1:2000) for 1 h, washed again, and developed using enhanced chemiluminescence. Protein signals were captured using a chemiluminescence imaging system and quantified with ImageJ or Bio-Rad Image Lab.

Immunofluorescence staining was performed on cultured cells, organoids, and tumor tissue sections. For cellular staining, cells grown on glass coverslips were fixed with 4% paraformaldehyde, permeabilized with 0.1–0.3% Triton X-100, and blocked with 5% BSA before overnight incubation at 4 °C with primary antibodies against GPX4 (Abcam, ab125066), SLC7A11 (Abcam, ab175186), 4-HNE (Abcam, ab46545), CRT (Abcam, ab2907), or HMGB1 (Abcam, ab79823). After washing, Alexa Fluor–conjugated secondary antibodies were applied for 1 h in the dark, followed by DAPI nuclear counterstaining and mounting in antifade medium.

### Patient-derived organoid culture

Human hepatocellular carcinoma (HCC) organoids were established from tumor tissues obtained from patients undergoing surgical resection at the Third Xiangya Hospital of Central South University. Written informed consent was obtained from all patients prior to sample collection. The study was approved by the Institutional Ethics Committee of the Third Xiangya Hospital, Central South University (Approval No. 2025-S116) and conducted in accordance with the Declaration of Helsinki.

Fresh tumor tissues were mechanically minced into small fragments and enzymatically digested with collagenase IV and dispase at 37 °C to generate single-cell suspensions. The resulting cell suspension was filtered through a 70 μm cell strainer, washed with cold PBS, and resuspended in growth factor–reduced Matrigel. Cells were then seeded as Matrigel domes in 24-well plates. After Matrigel polymerization, organoid culture medium consisting of advanced DMEM/F12 supplemented with B27, N2, EGF, Noggin, R-spondin1, and FGF10 was added. Organoids were maintained at 37 °C in a humidified incubator with 5% CO₂, with medium refreshed every 2–3 days. Organoids were passaged every 7–10 days by mechanical dissociation and replating in fresh Matrigel.

### Organoid ROS and γ-H2AX detection

ROS production and DNA damage in organoids were evaluated by confocal fluorescence imaging. For ROS detection, organoids were incubated with the fluorescent probe DCFH-DA (Beyotime) at a final concentration of 10 μM in serum-free medium at 37 °C for 30 min under live-cell conditions without permeabilization. After incubation, organoids were washed three times with PBS to remove excess dye. When required, nuclei were counterstained with Hoechst 33,342 (5 μg/mL) for 10 min at 37 °C. Fluorescence signals were observed and imaged using a confocal laser scanning microscope.

For γ-H2AX detection, organoids were first fixed with 4% paraformaldehyde for 20 min at room temperature, followed by permeabilization with 0.3% Triton X-100 for 10 min. Samples were then blocked with 5% bovine serum albumin (BSA) for 1 h and incubated with anti–γ-H2AX primary antibody (Abcam, ab81299) overnight at 4 °C. After washing with PBS, organoids were incubated with the corresponding fluorescent secondary antibody for 1 h at room temperature. Nuclei were counterstained with Hoechst 33,342, and fluorescence images were captured using confocal microscopy.

### CellROX staining and MitoSOX staining

Intracellular oxidative stress was assessed using CellROX Deep Red Reagent (Invitrogen, Thermo Fisher Scientific, Cat. No. C10422), and mitochondrial superoxide was detected using MitoSOX Red Mitochondrial Superoxide Indicator (Invitrogen, Thermo Fisher Scientific, Cat. No. M36008). After the indicated treatments, RR-HuH7 cells were incubated with CellROX Deep Red Reagent (5 μM, 30 min) or MitoSOX Red (5 μM, 10 min) at 37 °C in the dark, followed by PBS washing and Hoechst 33,342 nuclear staining. Fluorescence images were acquired using a confocal laser scanning microscope under identical imaging conditions.

### Co-culture assays

Bone marrow–derived dendritic cells (BMDCs) were generated from C57BL/6 mice and cultured in RPMI-1640 supplemented with 10% fetal bovine serum (FBS), GM-CSF (20 ng/mL), and IL-4 (10 ng/mL) for 6 days. Tumor cells were treated with BFMR-FA and/or irradiation as indicated. For co-culture experiments, a Transwell system with a 0.4-μm pore membrane (Corning) was used. BMDCs (1 × 10^5^ cells) were seeded in the lower chamber, and treated tumor cells (1 × 10^5^ cells) were placed in the upper insert. Cells were co-cultured in complete RPMI-1640 at 37 °C with 5% CO₂ for 24 h. After co-culture, BMDCs were collected from the lower chamber and analyzed for dendritic cell maturation markers by flow cytometry.

For DC–T cell co-culture assays, CD8⁺ T cells were isolated from mouse spleens using a CD8⁺ T cell isolation kit (Miltenyi Biotec) according to the manufacturer’s instructions. BMDCs that had undergone the indicated treatments were co-cultured with purified CD8⁺ T cells using a Transwell system (0.4 μm pore size) to allow indirect interaction through soluble factors. After co-culture, CD8⁺ T cells were harvested and analyzed for IFN-γ expression by flow cytometry.

### Immunofluorescence staining of tumor tissues

Tumor tissues were fixed, paraffin-embedded, sectioned, deparaffinized, and subjected to heat-induced antigen retrieval. After permeabilization and blocking, sections were incubated overnight at 4 °C with primary antibodies against Cleaved caspase-3 (Abcam, ab32042), CD86 (Abcam, ab239075), CD206 (Abcam, ab64693), or CD11b (Abcam, ab133357), followed by incubation with fluorophore-labeled secondary antibodies and DAPI staining. TUNEL staining was performed on parallel tumor sections using a commercial apoptosis detection kit according to the manufacturer’s instructions. All samples were imaged using a laser-scanning confocal microscope under identical acquisition parameters.

### In vitro and in vivo MRI T1-weighted imaging

The T1-weighted MRI performance of BFMR-FA was first evaluated using an in *vitro* columnar phantom. BFMR-FA was dispersed in PBS to prepare gradient concentrations of metal ions (Fe/Mn: 0, 10, 50, 100 μg/mL, calculated from ICP–MS measurements) and incubated for 30 min at pH 7.4, 6.5 and 5.5, corresponding to physiological conditions, the mildly acidic tumor microenvironment, and the lysosomal environment. Samples were then transferred to 1.5 mL EP tubes, placed in an MRI sample holder, and imaged using T1-weighted MRI.

In *vivo* MRI was performed in tumor-bearing mice carrying subcutaneous xenografts002E Mice were randomly assigned to BFMR or BFMR-FA groups and received intravenous injections of BFMR or BFMR-FA at an equivalent metal-ion dose (5 mg/kg). T1-weighted MRI scans were obtained at 0, 1, 2, 6, 12, 24 and 48 h post-injection to evaluate intratumoral accumulation kinetics and imaging enhancement.

### Flow cytometry analysis of tumor-infiltrating immune cells

Tumor tissues from different treatment groups were minced and digested in tumor dissociation buffer (BD Pharmingen, Cat: 554,593) at 37 °C for 1 h. The resulting single-cell suspensions were filtered through a 70 μm cell strainer. After incubation with FcγIII/IIR blocking antibodies to minimize nonspecific binding, the cells were stained with fluorophore-conjugated antibodies (1:200 dilution). CD45-Alexa700 (BD Pharmingen, Cat: 560,510) was used to identify total immune cells. Macrophage (TAM) polarization was analyzed using CD11b-BB515 (BD Pharmingen, Cat: 564,454), F4/80-BV421 (BD Pharmingen, Cat: 565,411), CD86-BV650 (BD Pharmingen, Cat: 564,200), and CD206-PECy7 (Thermo, Cat: 25–2061-82). DC maturation was assessed using CD11c-PE (eBioscience, Cat: 12–0114-82), CD80-APC (BioLegend, Cat: 104,713), and CD86-BV650 (BD Pharmingen, Cat: 564,200). T cell activation was determined using CD3-BV510 (BD Pharmingen, Cat: 563,024), CD4-BV605 (BD Pharmingen, Cat: 563,151), and CD8a-APC-Cy7 (BD Pharmingen, Cat: 557,654). All samples were analyzed by flow cytometry (FACSVerse, BD, USA), and FlowJo software was used to quantify immune cell subsets and functional status.

## Statistical analysis

All statistical analyses were conducted using GraphPad Prism 10.1.2. Quantitative data are reported as mean ± SD. Two-tailed, unpaired Student’s *t*-tests were used for two-group comparisons, and one-way or two-way ANOVA with Tukey’s post hoc test was used for multiple-group analyses. Normality and homogeneity of variance were verified before parametric testing. Statistical significance was defined as *p* < 0.05, with increasing significance denoted as *p* < 0.01, *p < 0.001, and **p < 0.0001. Unless otherwise stated, experiments were performed with at least three independent biological replicates.

## Results

### Construction and characterization of BFMR-FA

**Fig. 1:**
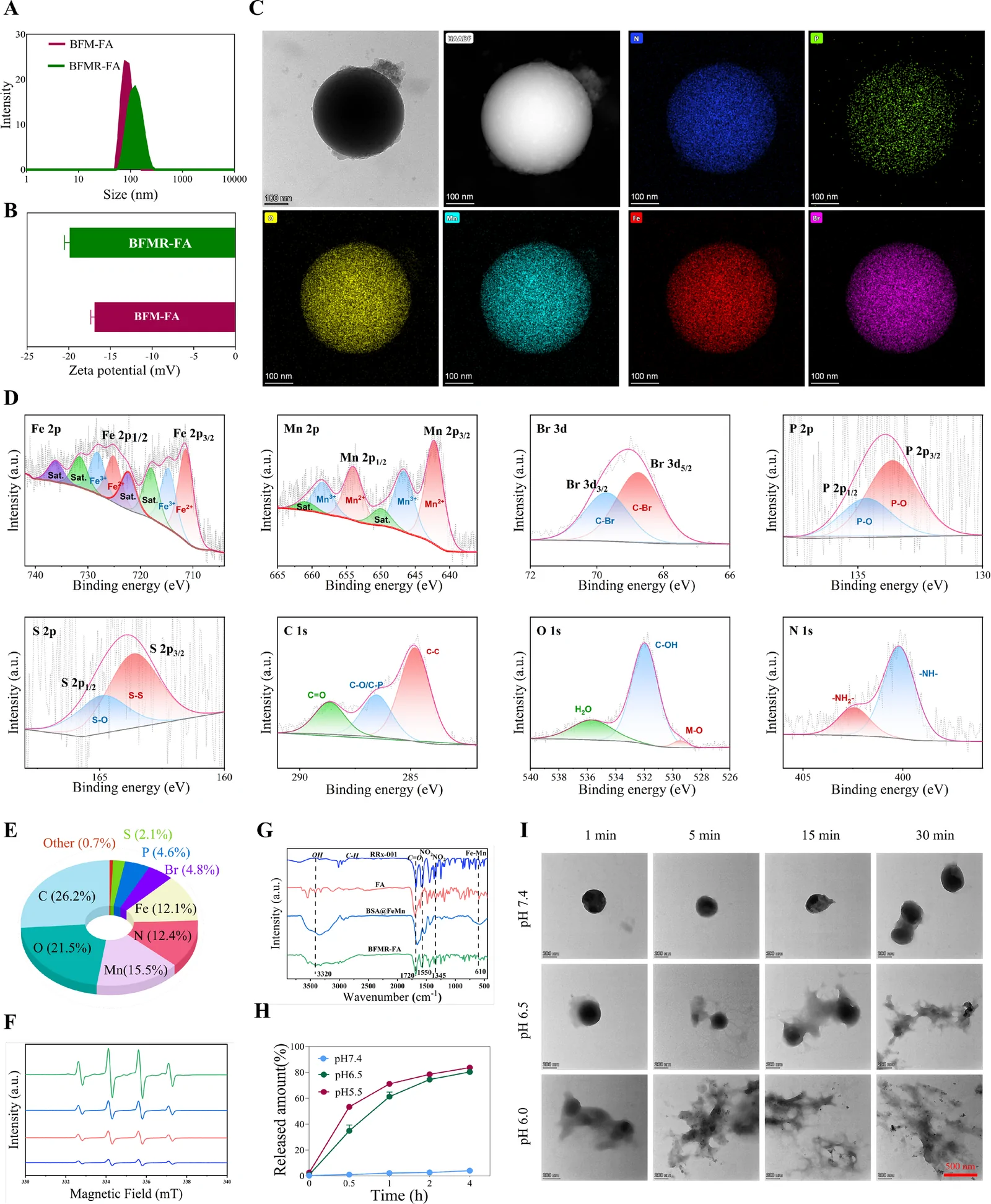
Construction and physicochemical characterization of BFMR-FA. **A** Dynamic light scattering (DLS) profiles showing the hydrodynamic size distributions of BFM-FA and BFMR-FA. **B** Zeta-potential measurements for both formulations. **C** Transmission electron microscopy (TEM) images illustrating the uniform spherical morphology of BFMR-FA. **D** XPS spectra confirming the elemental composition and oxidation states within the construct, Scale bar: 100 nm. **E** Semi-quantitative EDS mapping depicting the elemental ratios across the nanoparticle matrix. **F** EPR spectra showing free radical–related signals of the material. **G** FTIR spectra validating the integration of BSA, FA and RRx-001. **H** pH-responsive RRx-001 release profiles under physiological and acidic conditions. **I** TEM imaging reveals structural changes in BFMR-FA under different pH conditions, Scale bar: 500 nm

Using BSA as a biomolecular scaffold, Fe3⁺/Mn2⁺ as dual dopants, RRx-001 as a redox-active therapeutic payload, and folic acid (FA) as a targeting ligand, an acid-responsive radiosensitizing nanoplatform, designated BSA@FeMn@RRx-001-FA (BFMR-FA), was constructed. Systematic physicochemical characterization confirmed successful assembly of this multicomponent platform. Dynamic light scattering (DLS) revealed a narrow size distribution, with BFM-FA exhibiting an average hydrodynamic diameter of ~ 100 nm and BFMR-FA slightly increased to ~ 120 nm (Fig. 1A). Zeta-potential measurements showed a shift from − 17 mV (BFM-FA) to − 20 mV (BFMR-FA), indicating enhanced colloidal stability conferred by Fe/Mn co-doping and FA functionalization (Fig. 1B). TEM imaging demonstrated a uniform spherical morphology (Fig. 1C), while HAADF-STEM and elemental mapping confirmed the homogeneous distribution of Fe, Mn, Br, P, and S throughout the particle matrix, validating co-loading of all functional components.

UV–Vis spectroscopy (Fig. S1) further confirmed the successful incorporation of FA and RRx-001: BFMR-FA retained the characteristic FA absorption peak, the BSA protein band, and the subtle RRx-001 shoulder, with an overall increase in optical density, supporting efficient drug loading and surface modification. Optimization of the Fe:Mn molar ratio revealed a strong influence on encapsulation efficiency (EE) and loading efficiency (LE) (Fig. S2). Among the tested ratios, Fe:Mn = 1:1 provided the best balance between EE and LE. Consistently, nanoparticles synthesized at this ratio generated the strongest intracellular ROS signals (Fig. S3), indicating enhanced redox catalytic activity. Therefore, the 1:1 Fe/Mn ratio was selected for subsequent experiments.

XPS analyses (Fig. 1D) revealed multiple oxidation states—Fe2⁺/Fe3⁺ and Mn2⁺/Mn3⁺—consistent with redox-active catalytic behavior, while the Br 3 d signal confirmed RRx-001 loading. Binding energies for C, N, O, P, and S matched expected signatures from BSA and FA functional groups. Semi-quantitative EDS (Fig. 1E) showed excellent agreement between measured elemental ratios [C (26.2%), O (21.5%), Mn (15.5%), Fe (12.1%), N (12.4%), Br (4.8%), P (4.6%), S (2.1%)] and the designed composition. EPR spectra (Fig. 1F) displayed strong free-radical signals characteristic of Fe/Mn-mediated Fenton-like activity, confirming the system’s capacity for ·OH generation. FTIR analysis further confirmed the preservation of the BSA backbone peaks and the successful integration of FA and RRx-001 (Fig. 1G). Acid-responsive release studies showed minimal leakage of RRx-001 at pH 7.4. However, they markedly accelerated release at tumor-associated pH 6.5 and lysosomal pH 5.5 (Fig. 1H), consistent with TEM observations showing progressive particle collapse under acidic conditions (Fig. 1I).

### Enhanced radiosensitization validated in cellular and organoid systems

**Fig. 2:**
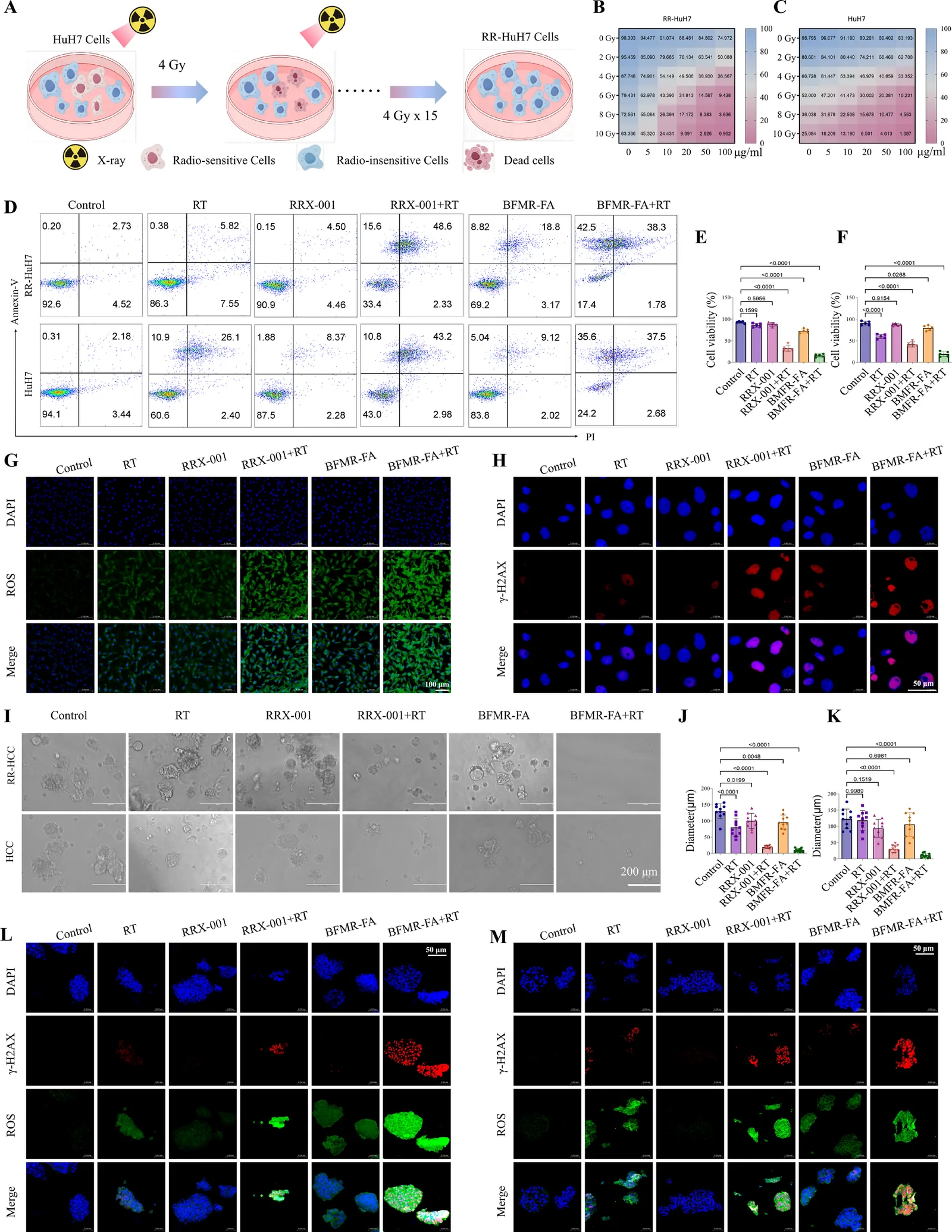
BFMR-FA markedly enhances the radiosensitivity of HCC cells and organoids. **A** Establishment of the RR-Huh7 radioresistant cell line through repeated fractionated irradiation. **B**, **C** Heatmaps showing the viability of Huh7 and RR-Huh7 cells across graded BFMR-FA concentrations and radiation doses (*n* = 5). **D**–**F** Flow cytometric quantification of apoptosis in both cell lines following the indicated treatments (*n* = 5). **G** DCFH-DA staining showing intracellular ROS levels in RR-Huh7 cells, Scale bar: 100 μm. **H** γ-H2AX immunofluorescence indicating radiation-induced DNA double-strand breaks, Scale bar: 50 μm. **I**–**K** Bright-field images depicting the effects of different treatments on radiosensitive and radioresistant HCC organoids, Scale bar: 200 μm, *n* = 10. **L**-**M** ROS and γ-H2AX staining illustrating treatment-dependent oxidative stress and DNA-damage responses in radioresistant organoids, Scale bar: 50 μm

To determine whether BFMR-FA can effectively augment radiotherapy in radioresistant hepatocellular carcinoma (HCC), we first established a stable RR-Huh7 radioresistant cell line through 15 rounds of fractionated irradiation at 4 Gy (radioresistant model development) (Fig. 2A). Viability heatmaps across BFMR-FA concentrations and radiation doses (Fig. 2B-C) confirmed a strong resistance phenotype: RR-Huh7 cells exhibited a pronounced resistance phenotype: even at 10 Gy, viability remained approximately 63%, whereas parental Huh7 cells decreased to ~ 25% at the same dose. Notably, combining BFMR-FA with irradiation markedly reduced viability in both RR-Huh7 and Huh7 cells, demonstrating that BFMR-FA can override intrinsic radioresistance and substantially enhance radiotherapeutic cytotoxicity.

Flow cytometric analysis of apoptosis confirmed these observations (Fig. 2D–F). In RR-Huh7 cells, irradiation alone induced minimal apoptosis, whereas both RRX-001 + RT and BFMR-FA + RT increased apoptosis, with BFMR-FA + RT producing the most pronounced effect. Consistent with this, DCFH-DA staining (Fig. 2G, Fig. S4A) showed that RT, RRX-001, or BFMR-FA alone generated only weak oxidative signals, whereas BFMR-FA + RT triggered a robust ROS surge. To validate this oxidative response, an independent probe, CellROX staining was performed (Fig. S5). It was found that BFMR-FA + RT induced the strongest CellROX fluorescence among all treatment groups, demonstrating that the enhanced oxidative stress was not dependent on a single ROS probe system. γ-H2AX immunofluorescence (Fig. 2H, Fig. S4B) further demonstrated that BFMR-FA + RT produced the highest levels of radiation-induced DNA double-strand breaks, underscoring its potent amplification of DNA damage as a mechanistic driver of radiosensitization.

To better capture tumor heterogeneity, we evaluated BFMR-FA in both radiosensitive (HCC) and radioresistant (RR-HCC) hepatocellular carcinoma organoid models. In HCC organoids, radiotherapy (RT) alone already produced a significant cytotoxic effect compared with the control group. In contrast, RR-HCC organoids showed minimal structural disruption following RT treatment, consistent with their radioresistant phenotype. Notably, BFMR-FA combined with RT induced pronounced collapse and shrinkage in both organoid models, indicating that BFMR-FA effectively enhances the response to radiotherapy across organoid systems (Fig. 2I–K). ROS and γ-H2AX staining corroborated these findings (Fig. 2L, 2M, Fig. S6), showing that BFMR-FA + RT elicited the strongest oxidative stress and DNA damage in both organoid types, with radioresistant organoids exhibiting a greater dependence on BFMR-FA for therapeutic sensitization. These results confirm that BFMR-FA maintains robust radiosensitizing activity even within complex three-dimensional tumor architectures.

Collectively, BFMR-FA markedly enhances HCC responsiveness to radiotherapy across monolayer cell models and patient-derived organoids. By amplifying oxidative stress and intensifying radiation-induced DNA damage, BFMR-FA effectively overcomes radioresistance and confers potent radiosensitizing activity.

### BFMR-FA enhances oxidative stress and lipid peroxidation in radioresistant

**Fig. 3:**
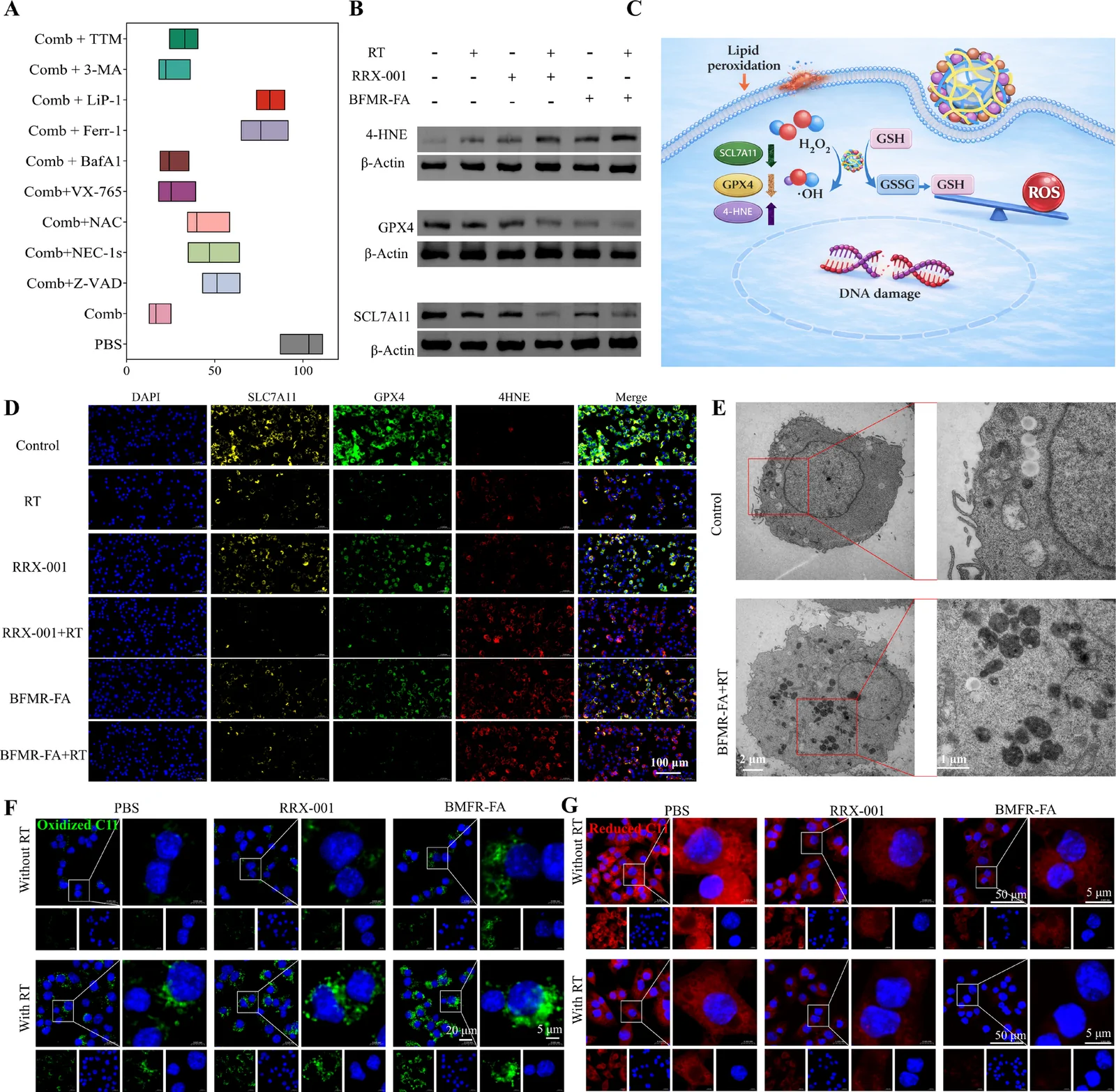
BFMR-FA enhances oxidative stress and lipid peroxidation in radioresistant RR-Huh7 cells.** A** Inhibitor rescue assay in RR-Huh7 cells. Comb denotes BFMR-FA combined with radiotherapy (RT). Cell viability was measured in the presence of different pathway inhibitors, (*n* = 5). **B** Western blot analysis of 4-HNE, GPX4, and SLC7A11 under the indicated treatments. **C** Proposed mechanism illustrating BFMR-FA–induced oxidative stress and redox imbalance. **D** Immunofluorescence staining of SLC7A11 (yellow), GPX4 (green), and 4-HNE (red). Nuclei were stained with DAPI (blue). Scale bar: 100 μm. **E** Transmission electron microscopy showing mitochondrial ultrastructural alterations after BFMR-FA + RT treatment. Scale bar: 2 μm or 1 μm. **F**–**G** C11-BODIPY staining showing increased oxidized C11 (green) and decreased reduced C11 (red), indicating enhanced lipid peroxidation

To define the mode of cell death induced by BFMR-FA in combination with radiotherapy, and to clarify how this treatment amplifies oxidative stress, we systematically examined multiple regulated cell death pathways in radioresistant RR-Huh7 cells treated with BFMR-FA (20 μM) with or without X-ray irradiation (4 Gy). In inhibitor rescue assays (Fig. 3A), cells were pretreated for 1 h with ferrostatin-1 (Fer-1, 10 μM), liproxstatin-1 (Lip-1, 100 nM), Z-VAD-FMK (30 μM), N-acetylcysteine (NAC, 5 mM), Necrostatin-1 (Nec-1, 10 μM), bafilomycin A1 (Baf-A1, 50 nM), or 3-methyladenine (3-MA, 5 mM) prior to treatment. Ferroptosis inhibitors, including Fer-1 and the lipid peroxidation blocker Lip-1, markedly attenuated BFMR-FA + RT-induced cytotoxicity, supporting the functional involvement of ferroptosis and lipid peroxidation. ROS scavenging with NAC, apoptosis inhibition with Z-VAD, and necroptosis inhibition with Nec-1 also provided varying degrees of protection, whereas autophagy inhibition showed a weaker protective effect. These results indicate that BFMR-FA + RT induces a ferroptosis-centered mixed oxidative cell death program, in which lipid peroxidation represents a major component, while apoptosis- and necroptosis-related pathways may also contribute to the overall cytotoxic response.

To further functionally validate the contribution of ferroptosis, GPX4-overexpressing cells (oeGPX4) were generated and subjected to BFMR-FA + RT treatment. Compared with wild-type cells, oeGPX4 HuH-7 and Hepa1-6 cells showed significantly higher viability after BFMR-FA + RT treatment (Fig. S7), indicating that GPX4 overexpression partially rescued tumor cells from the cytotoxic effect of the combined treatment. Consistently, clonogenic assays showed that oeGPX4 partially restored colony formation in both HuH-7 and Hepa1-6 cells following BFMR-FA + RT treatment (Fig. S8). These results provide additional functional evidence that ferroptosis contributes substantially to BFMR-FA + RT-induced tumor cell killing.

We next assessed how BFMR-FA influences redox homeostasis under different treatments (Fig. S9). Treatment with RRX-001 or BFMR-FA alone reduced the GSH/GSSG ratio and modestly increased malondialdehyde (MDA), indicating impaired antioxidant capacity. In contrast, BFMR-FA + RT induced the most profound GSH depletion, accompanied by robust MDA accumulation, indicating sustained lipid peroxidation. Consistent with these biochemical alterations, immunoblot analyses (Fig. 3B, Fig. S10) revealed increased expression of 4-HNE, a terminal lipid peroxidation adduct, in the combined treatment group, along with pronounced downregulation of GPX4 and SLC7A11. These findings indicate that BFMR-FA amplifies oxidative stress and perturbs cellular redox homeostasis (Fig. 3C).

Immunofluorescence staining further substantiated these molecular alterations (Fig. 3D, Fig. S11). Compared with control and single-treatment groups, BFMR-FA + RT markedly reduced SLC7A11 and GPX4 signals while increasing 4-HNE accumulation, consistent with the Western blot findings. Transmission electron microscopy provided additional ultrastructural evidence (Fig. 3E). Cells treated with BFMR-FA + RT displayed characteristic mitochondrial alterations, including reduced mitochondrial volume, increased membrane density, and disrupted or diminished cristae, whereas control cells maintained intact mitochondrial architecture. To further determine whether these mitochondrial structural changes were accompanied by metabolic alterations, Seahorse analysis was performed to assess cellular bioenergetics (Fig. S12). Compared with control cells, BFMR-FA + RT-treated cells showed reduced extracellular acidification rate (ECAR) and oxygen consumption rate (OCR), indicating impaired glycolytic acidification and mitochondrial respiration under combined treatment. Consistent with mitochondrial dysfunction, MitoSOX staining showed that BFMR-FA + RT markedly increased mitochondrial superoxide accumulation (Fig. S13). Lipid peroxidation was further evaluated using the C11-BODIPY probe (Fig. 3F–G, Fig. S14). BFMR-FA treatment increased the oxidized C11 signal, which was further enhanced following irradiation. Conversely, the reduced C11 signal was lowest in the BFMR-FA + RT group, indicating extensive lipid oxidation.

### BFMR-FA triggers STING signaling and promotes immunogenic cell death

**Fig. 4:**
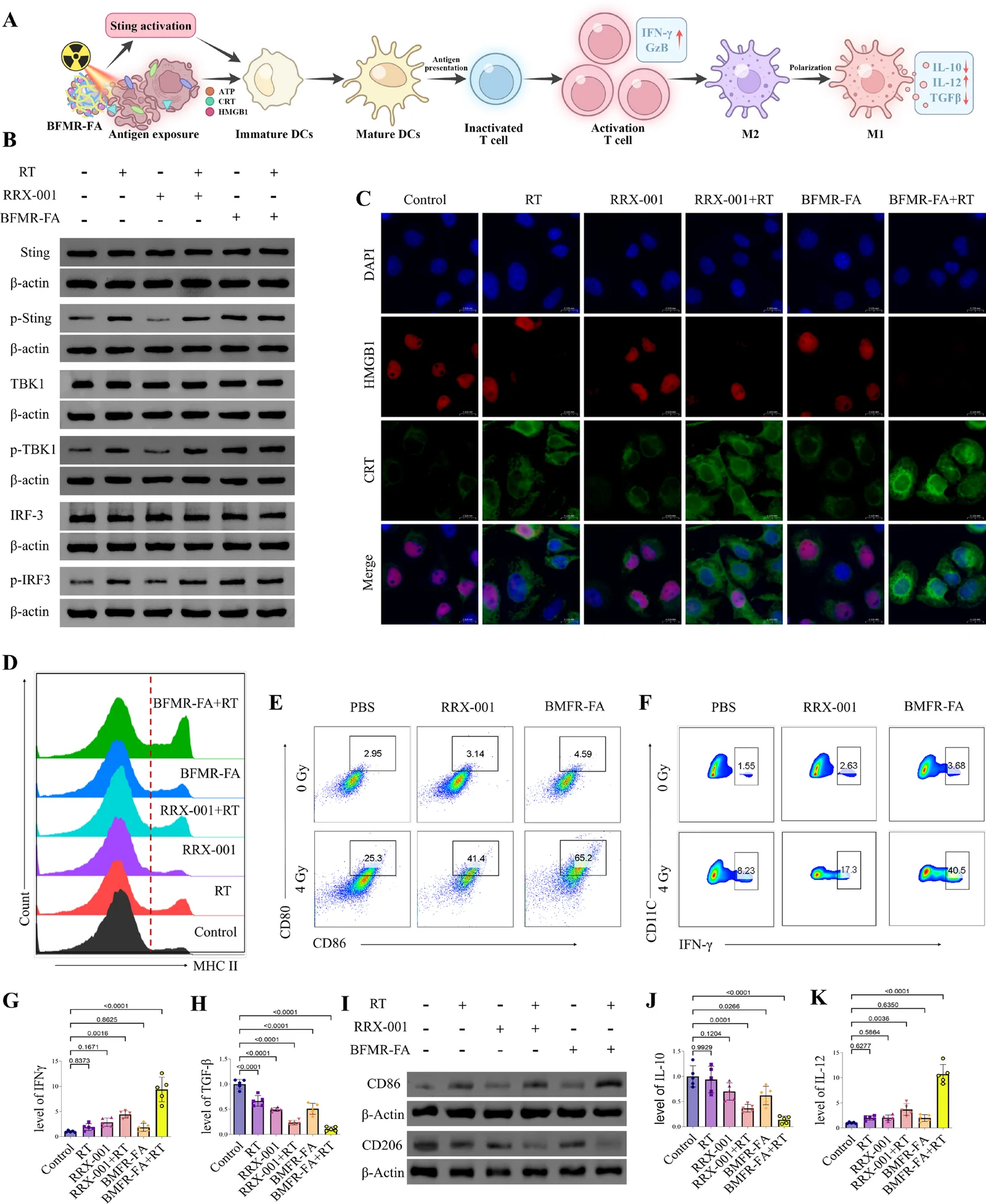
BFMR-FA activates STING signaling, induces ICD, and reprograms key immune effector populations. **A** Schematic illustration showing BFMR-FA–induced immunogenic cell death and STING activation, leading to dendritic cell (DC) maturation, T-cell activation, and macrophage polarization toward an M1 phenotype. **B** Western blot analysis of STING signaling pathway proteins (STING, p-STING, TBK1, p-TBK1, IRF3, and p-IRF3) under the indicated treatments. **C** Immunofluorescence staining of HMGB1 (red) and CRT (green) with DAPI nuclear staining (blue), showing increased DAMP exposure after BFMR-FA + RT treatment, Scale bar: 20 μm. **D** Flow cytometry histogram showing increased MHC-II expression in BMDCs after treatment. **E** Flow cytometry analysis of DC maturation markers CD80 and CD86 in BMDCs under the indicated conditions. **F** Flow cytometry analysis showing the proportion of IFN-γ⁺ cells within the CD8⁺ T-cell population. **G**–**H** ELISA analysis of IFN-γ and TGF-β secretion in the DC–T cell co-culture system (*n* = 5). **I** Western blot analysis of macrophage polarization markers CD86 (M1) and CD206 (M2). **J**–**K** ELISA measurement of macrophage cytokines IL-10 and IL-12 under different treatment conditions (*n* = 5)

To investigate how BFMR-FA enhances antitumor immune responses in radio-resistant hepatocellular carcinoma, we evaluated its effects on immunogenic stress signaling, STING pathway activation, dendritic cell maturation, T-cell activation, and macrophage polarization (Fig. 4A). Given that Mn^2^⁺ has been reported to potentiate cGAS–STING signaling by increasing the sensitivity of cGAS to cytosolic DNA and promoting downstream innate immune activation [32–34], we first examined whether the Mn-containing BFMR-FA platform could activate STING-associated signaling in radio-resistant HCC cells. Western blot analysis showed that BFMR-FA alone increased the phosphorylation levels of STING and TBK1 in RR-Huh7 cells, whereas the combination of BFMR-FA with radiotherapy induced the most pronounced activation of the STING–TBK1–IRF3 axis (Fig. 4B, Fig. S15), indicating that BFMR-FA further amplifies innate immune signaling under radiotherapy-induced stress.

Consistent with STING activation, BFMR-FA + RT also enhanced several hallmarks associated with immunogenic cell death (ICD). Immunofluorescence staining revealed increased CRT surface exposure and HMGB1 release in RR-Huh7 cells following BFMR-FA + RT treatment compared with radiotherapy or RRX-001 alone (Fig. 4C, Fig. S16). ELISA assays further confirmed elevated extracellular ATP release and HMGB1 secretion under BFMR-FA + RT treatment (Fig. S17). These observations indicate that BFMR-FA combined with radiotherapy can promote ICD-associated signals that may facilitate downstream antigen-presenting processes.

Given the increased release of danger-associated molecular patterns (DAMPs), we next evaluated dendritic cell activation. Flow cytometry analysis showed that BFMR-FA increased MHC-II expression in bone marrow–derived dendritic cells (BMDCs), whereas BFMR-FA combined with radiotherapy (RT) induced the most pronounced upregulation of MHC-II and the proportion of CD80⁺CD86⁺ mature DCs (Fig. 4D–E).

Furthermore, after co-culturing CD8⁺ T cells with DCs from different treatment groups, we observed a significant increase in the proportion of IFN-γ⁺ cells within the CD8⁺ T-cell population in the BFMR-FA + RT group (Fig. 4F), indicating enhanced cytotoxic T-cell activation. Consistently, IFN-γ secretion was increased, whereas TGF-β levels were reduced in the culture supernatant (Fig. 4G–H), suggesting a shift toward a pro-inflammatory effector T-cell response.

We further explored whether BFMR-FA + RT could influence macrophage polarization. Western blot and flow cytometry analyses showed that macrophages co-cultured with DCs conditioned by BFMR-FA or BFMR-FA + RT exhibited reduced expression of the M2-associated marker CD206 and increased expression of the M1-associated marker CD86 (Fig. 4I, Fig. S18), suggesting a shift toward an M1-like phenotype in this in vitro system. ELISA results were consistent with this observation, showing reduced secretion of IL-10 (Fig. 4J) and increased levels of IL-12 (Fig. 4K) in macrophages exposed to BFMR-FA + RT–conditioned signals.

Taken together, these results suggest that BFMR-FA combined with radiotherapy may promote a series of immune-related responses in RR-Huh7 cells, including induction of ICD-associated signals, activation of STING signaling, promotion of DC maturation, enhanced T-cell activation, and macrophage polarization toward an M1-like phenotype in the co-culture system. These findings provide potential mechanistic insight into the radiosensitizing effects of BFMR-FA.

### BFMR-FA enables targeted delivery and enhanced MRI visualization

To verify whether FA modification enhances tumor-cell uptake, we first performed in vitro competitive uptake assays using Cy5.5-labeled nanoparticles (Fig. S19). Compared with non-targeted BFMR, BFMR-FA showed markedly stronger intracellular fluorescence in HuH-7, RR-HuH-7, Hepa1-6, and RR-Hepa1-6 cells. Importantly, pretreatment with excess free FA substantially reduced BFMR-FA uptake, supporting FA-mediated cellular internalization. In contrast, no obvious difference in nanoparticle uptake was observed among BFMR, BFMR-FA, and FA-pretreated BFMR-FA groups in nonmalignant LO2 and AML-12 cells, indicating limited FA-mediated enrichment in normal liver cells. These results confirm the tumor-cell-preferential uptake of BFMR-FA and support the rationale for FA-mediated targeting.

**Fig. 5:**
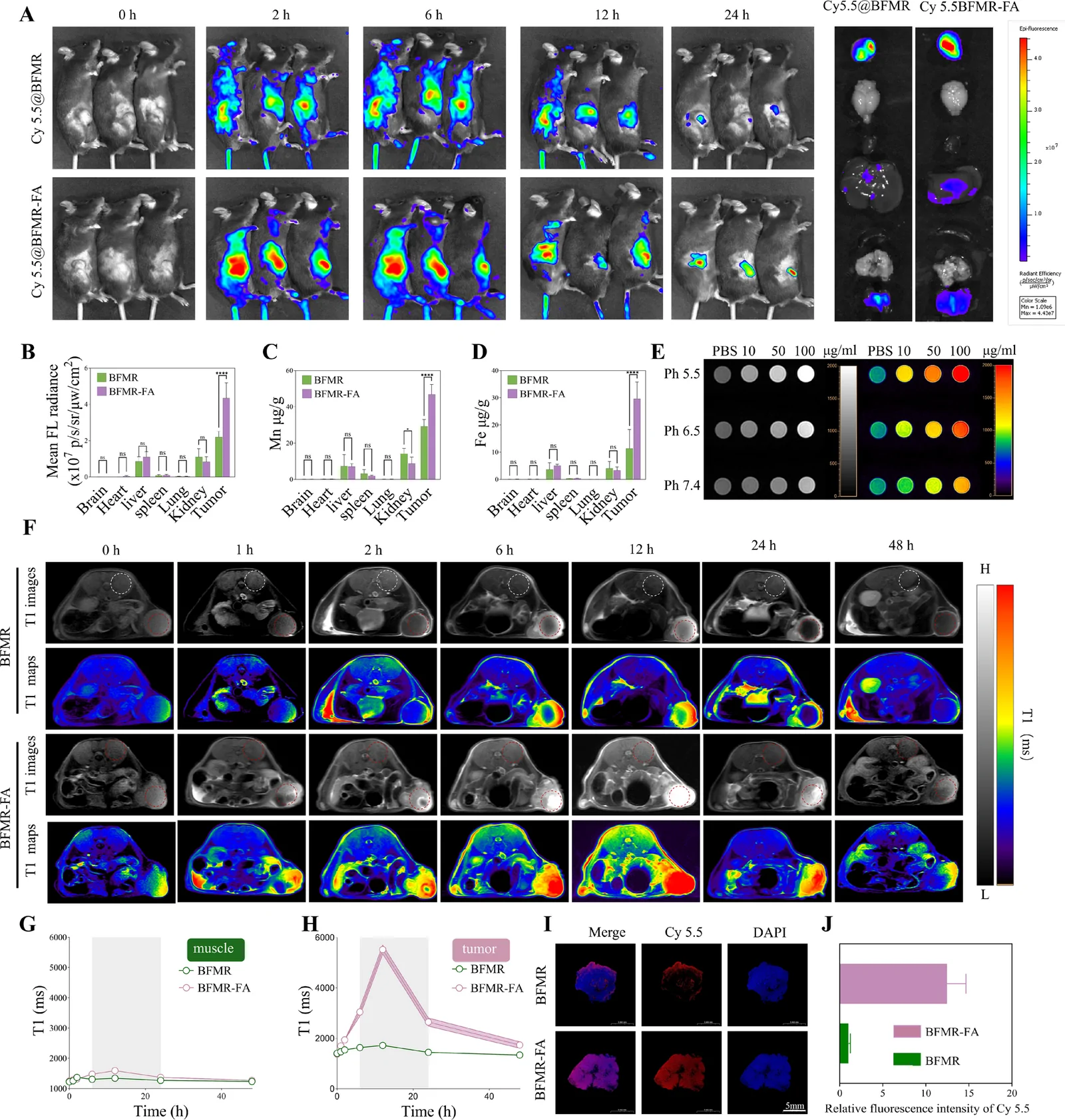
BFMR-FA demonstrates enhanced tumor accumulation and superior multimodal imaging capability. **A** Whole-body fluorescence imaging of tumor-bearing mice after intravenous injection of Cy5.5-labeled BFMR or BFMR-FA at the indicated time points. Representative ex vivo fluorescence images of major organs and tumors are shown on the right. **B** Quantification of mean fluorescence intensity in major organs and tumors (*n* = 5). **C**, **D** ICP–MS quantification of Mn and Fe accumulation in tissues after treatment with BFMR or BFMR-FA (*n* = 5). **E**) In vitro T1-weighted MR images of BFMR-FA at different concentrations under pH 7.4, 6.5, and 5.5 conditions. **F** In vivo T1-weighted MRI and corresponding T1 maps of tumor-bearing mice after administration of BFMR or BFMR-FA at the indicated time points. **G**–**H** Quantification of T1 relaxation times in muscle and tumor tissues over time (*n* = 5). **I** Fluorescence imaging of tumor sections showing Cy5.5 distribution in tumors treated with BFMR or BFMR-FA. Nuclei were stained with DAPI, Scale bar: 5 mm. **J** Quantification of relative Cy5.5 fluorescence intensity in tumor tissues (*n* = 5)

To comprehensively assess the in vivo behavior and multimodal imaging performance of BFMR-FA, we next performed whole-body fluorescence imaging using Cy5.5-labeled nanoparticles (Fig. 5A). Compared with unmodified BFMR, Cy5.5–BFMR-FA produced markedly stronger and more spatially confined tumor fluorescence as early as 2–6 h post-injection, reaching a pronounced peak at 12–24 h. In contrast, Cy5.5–BFMR exhibited weaker and more diffuse signals, indicating less efficient tumor accumulation. Ex vivo fluorescence imaging further confirmed substantially higher Cy5.5–BFMR-FA deposition in tumor tissues.

ICP–MS quantification (Fig. 5B–D) further supported these imaging findings. Mn and Fe levels in tumors were several-fold higher in the BFMR-FA group, whereas differences in major organs, including the liver, spleen, and kidney, remained minimal. These results indicate that FA modification enhances tumor-targeted delivery while maintaining a favorable systemic biodistribution profile.

We next assessed MRI contrast performance. In vitro T1 mapping (Fig. 5E) showed that BFMR-FA produced concentration-dependent T1 signal enhancement across pH 7.4, 6.5, and 5.5. Notably, T1 contrast increased substantially under mildly acidic conditions, consistent with pH-responsive structural disassembly and enhanced Mn/Fe ion release in tumor-like microenvironments.

In vivo MRI (Fig. 5F) further validated these imaging properties. BFMR-FA produced pronounced tumor T1 signal enhancement within 1 h after injection, peaking at 6–12 h, whereas BFMR generated weaker and more transient signals. Importantly, BFMR-FA induced minimal T1 changes in muscle tissue (Fig. 5G), further supporting its tumor-preferential accumulation and prolonged intratumoral retention.

Fluorescence imaging of tumor sections (Fig. 5I–J, Fig. S20) provided cellular-level confirmation of these observations. Cy5.5–BFMR-FA exhibited markedly greater intracellular accumulation than BFMR, with strong punctate fluorescence distributed throughout tumor tissues, indicating superior tumor targeting and intratumoral penetration.

Collectively, these results demonstrate that BFMR-FA enables FA-mediated tumor-cell uptake, enhanced intratumoral accumulation, and robust T1 MRI contrast both in vitro and in vivo. This integrated targeting and imaging capability supports real-time visualization of nanoparticle delivery and provides a strong basis for image-guided radiosensitization.

### BFMR-FA enhances in vivo radiosensitization by promoting ferroptosis

**Fig. 6:**
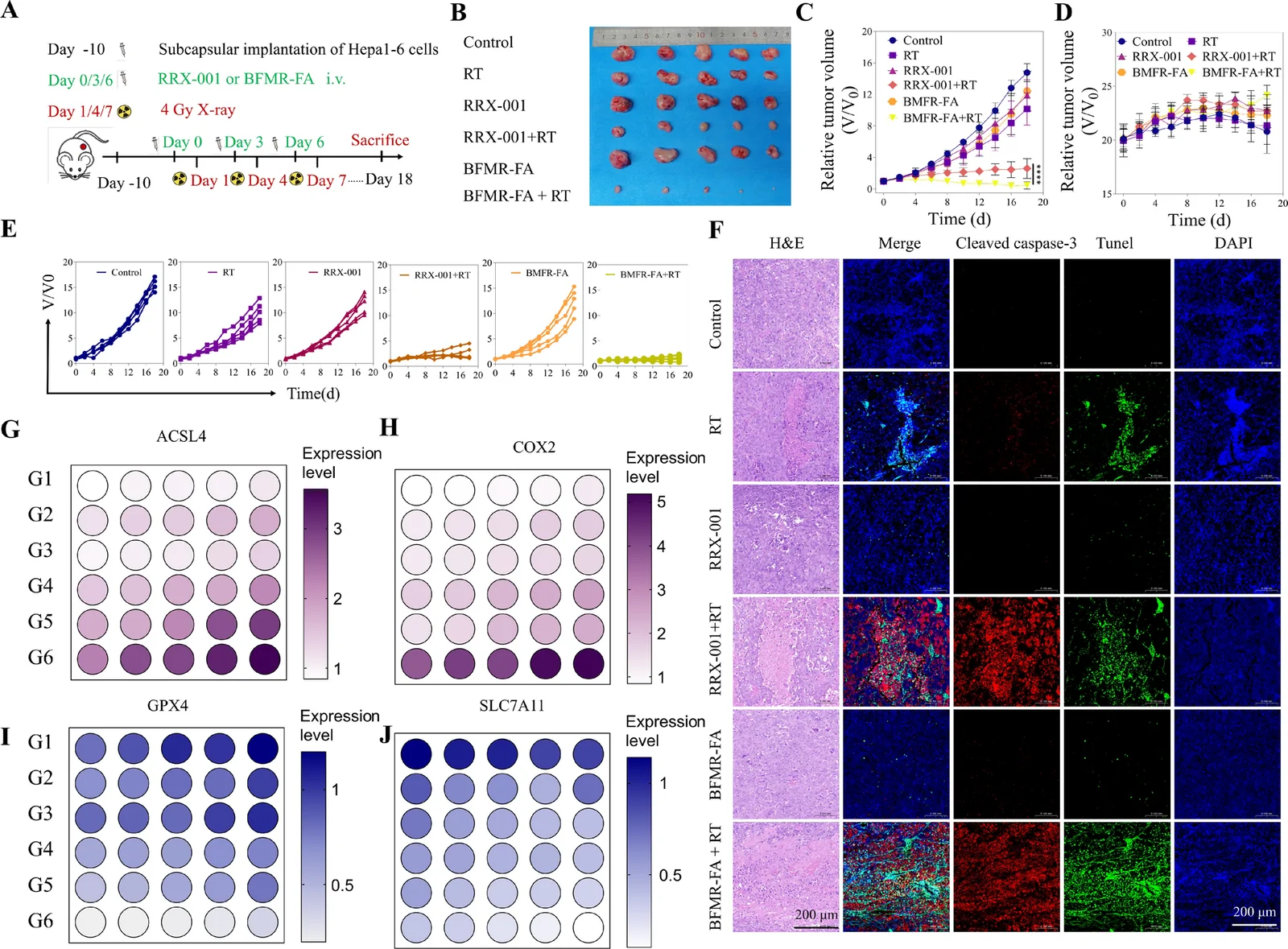
BFMR-FA combined with radiotherapy suppresses tumor growth in vivo**.** **A** Schematic illustration of the treatment schedule in the RR-Huh7 tumor-bearing mouse model. Mice received intravenous administration of RRX-001 or BFMR-FA on days 0, 3, and 6, followed by X-ray irradiation (4 Gy) on days 1, 4, and 7. **B** Representative images of excised tumors from different treatment groups at the Day 18. **C** Relative tumor growth curves of mice receiving different treatments. **D** Quantification of relative tumor volume in each group (*n* = 5). **E** Individual tumor growth curves for each treatment group, showing detailed tumor growth dynamics in control, RT, RRX-001, RRX-001 + RT, BFMR-FA, and BFMR-FA + RT groups. **F** Histological and immunofluorescence analyses of tumor sections, including H&E staining, cleaved caspase-3 staining, and TUNEL assay, indicating increased apoptosis in the BFMR-FA + RT group, Scale bar: 200 μm. **G**–**J** mRNA expression levels of ferroptosis-related genes ACSL4, COX2, GPX4, and SLC7A11 in tumor tissues from different treatment groups (*n* = 5). G1–G6 represent Control, RT, RRX-001, RRX-001 + RT, BFMR-FA, and BFMR-FA + RT, respectively

To assess the in *vivo* therapeutic efficacy of BFMR-FA, we established a radioresistant RR-HuH7 xenograft model following the treatment schedule outlined in Fig. 6A. Mice received intravenous RRX-001 or BFMR-FA on Days 0, 3 and 6, followed by 4 Gy irradiation on Days 1, 4 and 7—each administered 24 h after nanoparticle injection. Tumor growth and body weight were monitored throughout the study to evaluate both antitumor activity and systemic tolerability.

A clear hierarchy of therapeutic outcomes emerged across treatment groups (Fig. 6B–E). Tumors were harvested and photographed at the experimental endpoint (Day 18, n = 5 per group), as shown in Fig. 6B. Tumors in the control cohort grew rapidly and continuously. RT alone, RRX-001 monotherapy, and BFMR-FA monotherapy produced only modest growth delay, indicating that neither oxidative-stress amplification (RRX-001), catalytic ROS generation (BFMR-FA), nor standard irradiation is sufficient to overcome radioresistance. The RRX-001 + RT combination partially restored radiosensitivity, resulting in a noticeable, but still limited, suppression of tumor growth (Fig. 6C and Fig. 6E). In contrast, the BFMR-FA + RT group exhibited near-complete tumor growth arrest, with several xenografts showing overt regression and no significant weight loss, highlighting the radiosensitizing effect of BFMR-FA + RT (Fig. 6D).

Histopathological and molecular analyses (Fig. 6F, Fig. S21) corroborate the superior therapeutic response of BFMR-FA + RT. H&E staining showed the most extensive coagulative necrosis in the combination group. Cleaved caspase-3 and TUNEL staining followed a hierarchical pattern, minimal in monotherapies, increased in RRX-001 + RT, and maximal in BFMR-FA + RT, indicating robust activation of apoptotic pathways under dual treatment.

To further assess ferroptosis involvement in vivo, qPCR analysis was performed to examine the expression of key lipid peroxidation regulators (Fig. 6G–J). BFMR-FA + RT markedly increased the pro-ferroptotic genes ACSL4 and COX2, while significantly suppressing the ferroptosis-protective genes GPX4 and SLC7A11, with stronger effects than RRX-001 + RT or either monotherapy. These transcriptional changes are consistent with enhanced lipid peroxidation and disruption of antioxidant defenses in tumor tissues.

Together, these data establish that BFMR-FA + RT induces deep, multifaceted tumor destruction in radioresistant HCC by simultaneously amplifying apoptosis and ferroptosis, and achieves a level of tumor control unattainable with either RT alone or conventional radiosensitizers.

### BFMR-FA reprograms tumor immunity to overcome immune suppression

**Fig. 7:**
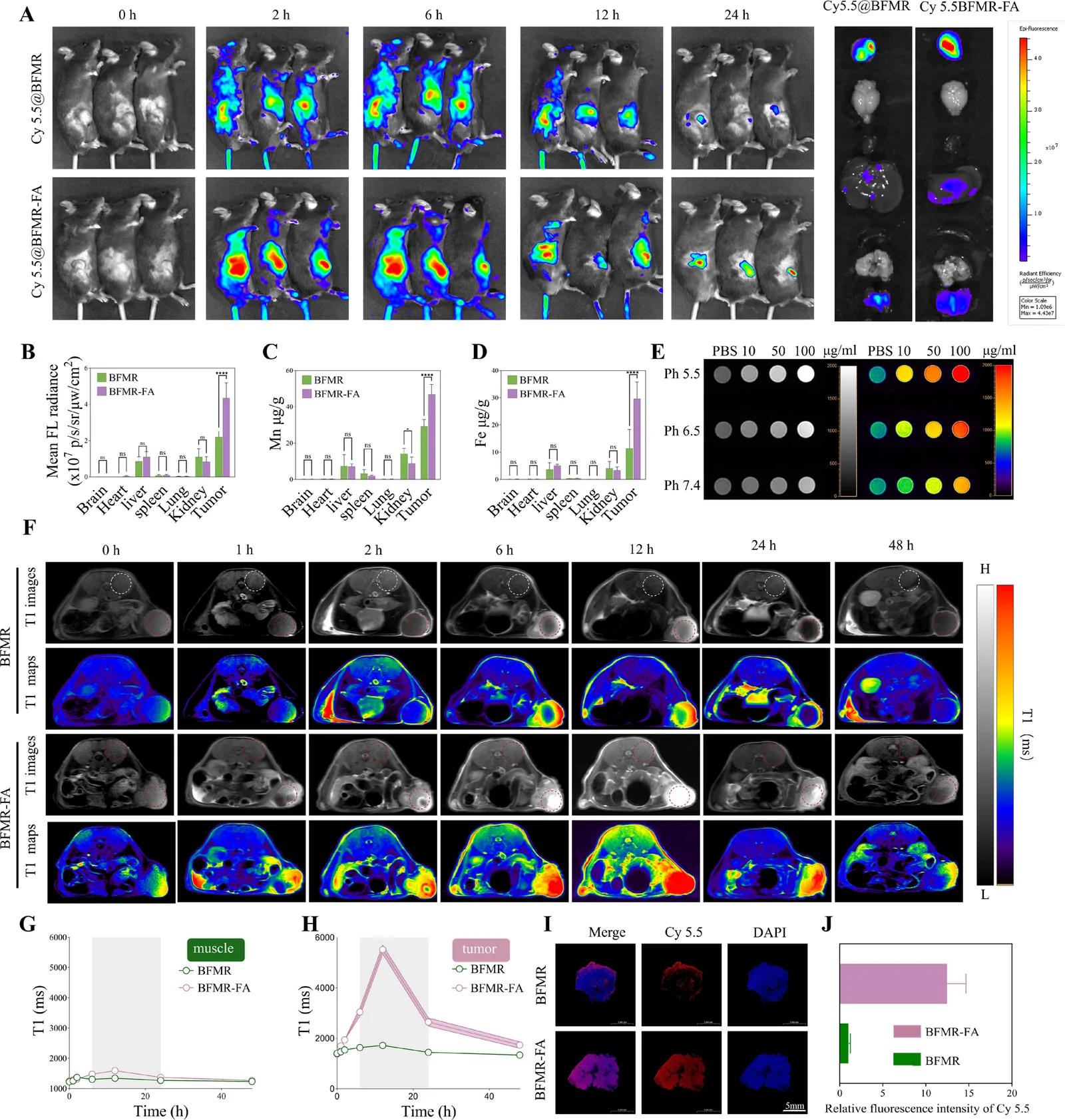
BFMR-FA reprograms the immune microenvironment in an orthotopic radioresistant HCC model. **A** Experimental schedule of the orthotopic RR-Hepa1-6 liver tumor model. Mice received intravenous administration of RRX-001 or BFMR-FA on days 0, 2, and 4, followed by X-ray irradiation (4 Gy) on days 1 and 4. **B** Representative images of tumor-bearing livers from different treatment groups at the experimental endpoint. **C** Quantification of liver weight/body weight (LW/BW) ratios in each group (*n* = 5). **D**–**E** Flow cytometry analysis and quantification of mature dendritic cells (CD80⁺CD86⁺) in tumor tissues (*n* = 5). **F**, **G** Representative flow cytometry plots and quantification of CD8⁺ T cells within CD3⁺ T cells in the tumor microenvironment (*n* = 5). **H**, **I** Flow cytometry analysis and quantification of macrophage polarization, showing the proportion of CD86⁺ (M1-like) macrophages within F4/80⁺CD11b⁺ cells (*n* = 5). **J** Immunofluorescence staining of tumor sections showing CD11b (red), CD86 (yellow), and CD206 (green), with nuclei stained by DAPI, Scale bar: 100 μm. **K** Heatmap showing cytokine levels (IFN-γ, TNF-α, GzB, TGF-β, IL-12, and IL-10) in tumor tissues across different treatment groups (*n* = 6)

Because immunosuppression is commonly observed in radioresistant tumors, we next examined whether BFMR-FA treatment was associated with changes in the immune contexture in an orthotopic RR-Hepa1-6 hepatocellular carcinoma model treated according to the indicated protocol (Fig. 7A). Macroscopic examination of excised tumors suggested that the combination of BFMR-FA and radiotherapy resulted in a greater reduction in intrahepatic tumor burden compared with RT, RRX-001, or BFMR-FA alone (Fig. 7B). Consistently, the liver-to-body weight ratio was lower in the BFMR-FA + RT group relative to the other treatment groups (Fig. 7C).

Flow cytometry analysis indicated increased antigen-presenting activity within the tumor microenvironment. Specifically, the proportion of CD80⁺CD86⁺ dendritic cells among CD11c⁺ cells was higher in tumors from the BFMR-FA + RT group compared with the other groups (Fig. 7D, E). In parallel, analysis of tumor-infiltrating T cells showed an increased proportion of CD8⁺ T cells among CD3⁺ T cells following BFMR-FA + RT treatment (Fig. 7F, G).

Macrophage phenotypes within tumors were also examined. Flow cytometry analysis showed a reduction in CD206⁺ macrophages together with an increase in CD86⁺ macrophages within the CD11b⁺F4/80⁺ population in the BFMR-FA + RT group (Fig. 7H, I), suggesting a shift toward a more pro-inflammatory macrophage phenotype.

Immunofluorescence staining provided spatial evidence consistent with these observations, showing increased CD11b⁺ and CD86⁺ signals together with reduced CD206⁺ staining in tumor tissues from the BFMR-FA + RT group (Fig. 7J, Fig. S22). Cytokine profiling further indicated higher levels of IFN-γ, TNF-α, Granzyme B, and IL-12, accompanied by relatively lower levels of TGF-β and IL-10 (Fig. 7K).

Taken together, these findings suggest that BFMR-FA combined with radiotherapy is associated with changes in several immune cell populations and cytokine profiles within the tumor microenvironment in vivo.

### Biosafety evaluation of BFMR-FA

**Fig. 8:**
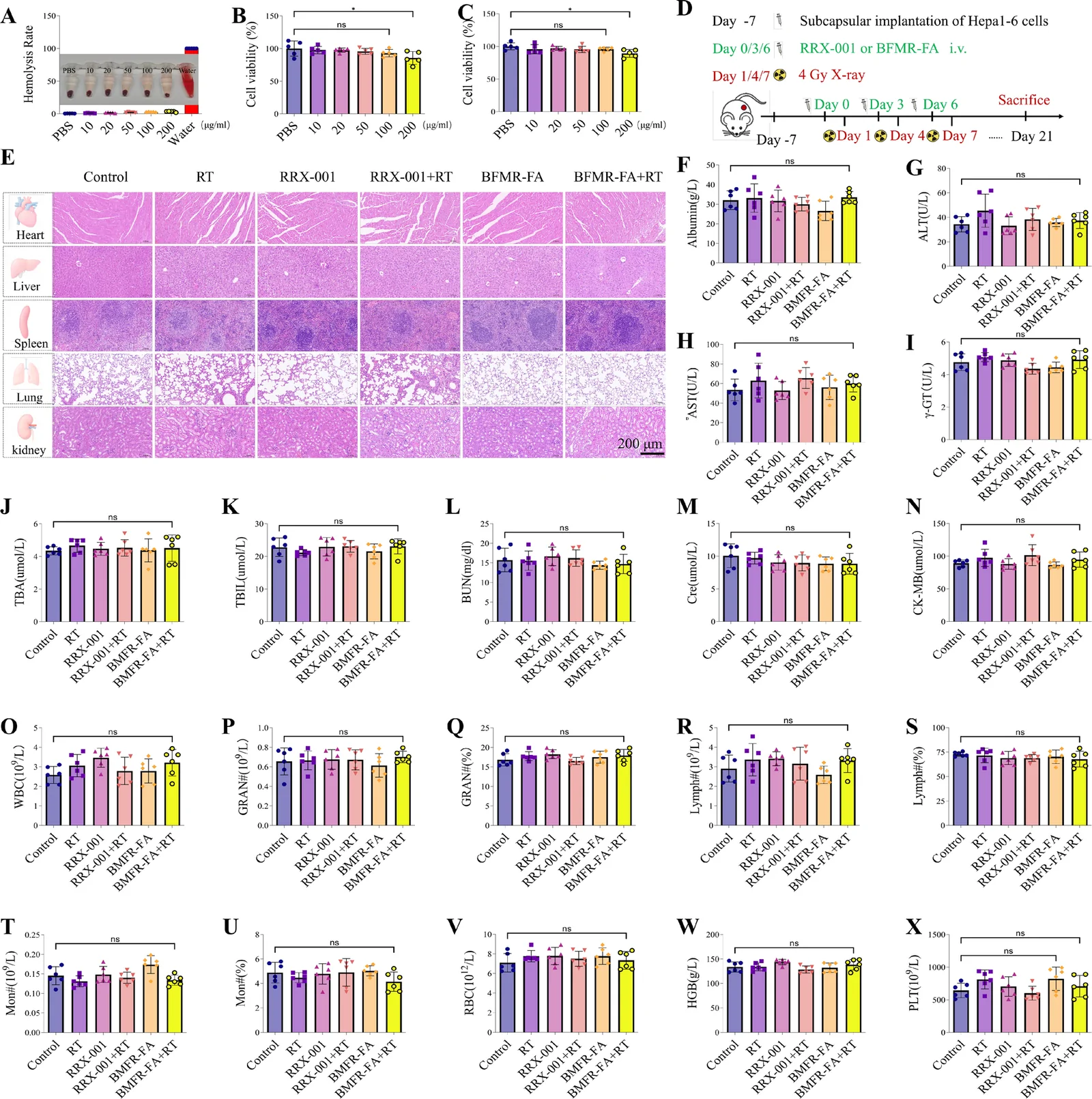
Comprehensive biosafety evaluation of BFMR-FA. **A** Hemolysis assessment of BFMR-FA compatibility with erythrocytes (n = 5). Cell viability assays evaluating the cytotoxicity of BFMR-FA toward (B) AML12 hepatocytes and (**C**) HUVECs (*n* = 5). **D** Treatment schedule for in *vivo* biosafety analysis (*n* = 5). **E** Representative H&E staining of major organs in each treatment group, Scale bar: 200 μm. **F**–**N** Serum biochemistry profiling of hepatic, renal and cardiac function indices following treatment (n = 5). (O–X) Hematological parameters assessing systemic tolerance across groups (n = 5)

Ensuring that radiosensitization strategies have not only therapeutic potency but also a predictable, well-tolerated safety profile is essential for clinical translation. Accordingly, we conducted a comprehensive assessment of the biosafety of BFMR-FA across hematologic, cellular, and systemic levels. Hemolysis testing (Fig. 8A) showed that BFMR-FA induced negligible erythrocyte disruption across a broad concentration range (10–200 μg/mL), with hemolysis ratios indistinguishable from those of PBS controls, indicating excellent blood compatibility and supporting its suitability for intravenous administration. In parallel, cytotoxicity assays in normal murine hepatocytes (AML12) and HUVECs (Fig. 8B–C) showed that BFMR-FA maintained > 85% cell viability even at high exposure levels, underscoring its minimal off-target toxicity toward nonmalignant tissues.

At the in vivo level, tumor-bearing C57BL/6 mice implanted with Hepa1-6 orthotopic liver tumors were treated according to the regimen described above and sacrificed on Day 18 after treatment initiation (Fig. 8D). Histopathological examination of major organs (heart, liver, spleen, lungs, and kidneys) (Fig. 8E) revealed preserved tissue architecture in all BFMR-FA–treated groups, with no evidence of inflammatory infiltration, necrosis, or fibrotic remodeling, closely resembling the control animals. Serum biochemistry (Fig. 8F–N) further confirmed the functional stability of the hepatic, renal, and cardiac systems, with albumin, transaminases, γ-GT, BUN, creatinine, and CK-MB remaining within the physiological range. Likewise, complete blood count parameters (Fig. 8O–X) remained stable across treatment groups, with leukocyte, erythrocyte, and platelet indices showing no deviation from baseline hematologic homeostasis.

Collectively, these findings demonstrate that BFMR-FA exhibits high systemic tolerability, preserving hematologic integrity, organ structure, and metabolic function while delivering potent radiosensitization in tumors. The convergence of robust efficacy and favorable biosafety provides a solid foundation for advancing BFMR-FA toward clinically actionable radiosensitization strategies.

## Discussion

Radioresistance in hepatocellular carcinoma (HCC) is not simply a consequence of insufficient radiation-induced DNA damage [35, 36], but reflects a coordinated adaptive state sustained by redox buffering, lipid peroxide detoxification, DNA damage repair, and immune suppression [37–40]. This layered defense architecture allows tumor cells to buffer transient oxidative insults, repair sublethal injury, and remain immunologically silent after irradiation. Therefore, effective radiosensitization may require more than amplification of radiation damage alone; it may require disruption of the homeostatic networks that determine whether radiation-induced stress remains reversible or is converted into lethal injury. In this study, BFMR-FA was developed as an integrated nanotherapeutic platform combining Fe/Mn-mediated redox catalysis, RRX-001-mediated thiol reactivity, FA-mediated tumor-cell uptake, and MRI visibility. The resulting findings support a proposed oxidative homeostasis collapse–driven radio-immuno synergy (OHC-RIS) model, in which redox destabilization and immune activation cooperate to attenuate radioresistance.

A central finding of this study is that BFMR-FA intensifies radiation-induced oxidative injury by simultaneously increasing redox pressure and weakening antioxidant buffering. In radioresistant tumor cells, radiation-induced ROS can be partially counteracted by glutathione-dependent antioxidant defenses and GPX4-mediated lipid peroxide detoxification [41, 42]. BFMR-FA interferes with this protective state through its Fe/Mn redox-active components and RRX-001-mediated thiol reactivity. Consistently, BFMR-FA + RT reduced the GSH/GSSG ratio, increased MDA and 4-HNE accumulation, suppressed GPX4 and SLC7A11, and enhanced C11-BODIPY oxidation. These findings indicate that BFMR-FA does not merely increase ROS levels, but promotes sustained lipid peroxidation by disrupting the redox-buffering capacity of radioresistant HCC cells under irradiation.

The cell death pattern induced by BFMR-FA + RT is best interpreted as a ferroptosis-centered mixed oxidative cell death program. Multiple lines of evidence support ferroptosis involvement, including lipid peroxidation markers, ferroptosis inhibitor rescue, suppression of GPX4/SLC7A11, upregulation of ACSL4/COX2, and the protective effect of GPX4 overexpression. The oeGPX4 rescue experiments are particularly important because they provide functional evidence that restoration of a key ferroptosis-suppressive enzyme attenuates BFMR-FA + RT-induced tumor cell killing. However, the inhibitor rescue data also indicate that ferroptosis is not the only process engaged. NAC, Z-VAD, and Necrostatin-1 provided varying degrees of protection, suggesting that ROS-dependent injury, apoptotic signaling, and necroptosis-related pathways may also contribute to the overall cytotoxic response. This convergence is biologically meaningful: under severe oxidative stress, regulated death pathways may not operate as isolated modules, but rather as interconnected execution programs shaped by mitochondrial injury, membrane damage, lipid oxidation, and failure of antioxidant recovery. Therefore, the therapeutic strength of BFMR-FA + RT may arise not from exclusive activation of one death pathway, but from forcing radioresistant cells into a composite oxidative death state that is difficult to adaptively escape.

The mitochondrial findings further support this interpretation. TEM revealed mitochondrial shrinkage, increased membrane density, and disrupted cristae after BFMR-FA + RT treatment, consistent with oxidative mitochondrial stress. Consistently, Seahorse analysis revealed concomitant reductions in ECAR and OCR after BFMR-FA + RT treatment, suggesting a broad impairment of cellular bioenergetic activity, including both glycolytic acidification and mitochondrial respiration. In parallel, MitoSOX staining demonstrated increased mitochondrial superoxide accumulation, suggesting that mitochondrial ROS contributes to the broader oxidative injury induced by BFMR-FA + RT. Together, these results indicate that mitochondrial dysfunction is an integral component of the oxidative collapse induced by the combined treatment.

Beyond tumor cell killing, the present study suggests that oxidative damage is coupled to local immune activation. BFMR-FA + RT promoted several hallmarks of immunogenic cell death, including calreticulin exposure, HMGB1 release, and ATP secretion [43, 44]. These danger-associated signals were accompanied by STING–TBK1–IRF3 activation, dendritic cell maturation, enhanced CD8⁺ T-cell activation, macrophage polarization toward a pro-inflammatory phenotype, and a cytokine profile favorable to antitumor immunity in the orthotopic immunocompetent model. These findings support a model in which redox destabilization not only enhances cytotoxicity but also increases the immunologic visibility of irradiated tumor cells. The STING–TBK1–IRF3 axis may participate in this process and, together with ICD-associated danger signal release and DC maturation, contribute to local immune remodeling after radiotherapy.

Another important contribution of this work is the integration of therapeutic function with imaging traceability. The Fe/Mn composition confers T1-weighted MRI contrast, enabling noninvasive visualization of intratumoral accumulation and pH-responsive behavior. Together with fluorescence imaging and ICP–MS quantification, the MRI results demonstrate that BFMR-FA achieves more efficient tumor accumulation than non-targeted BFMR. The FA competitive uptake experiments further strengthen the targeting rationale by showing that BFMR-FA uptake is enhanced in HCC and radioresistant HCC cells and can be reduced by excess free FA, whereas uptake differences are limited in nonmalignant liver cells. This tumor-preferential uptake provides an important explanation for why strong oxidative amplification can be achieved in tumors while maintaining acceptable systemic tolerability. In this context, MRI visibility is not merely an imaging feature, but provides a translationally relevant means to monitor delivery, guide therapeutic timing, and support image-guided radiosensitization.

Safety remains a central challenge for any ROS-amplifying strategy [45]. This issue is particularly important here because Fe/Mn-mediated catalysis, RRX-001-mediated thiol disruption, and radiation-induced water radiolysis could theoretically injure normal tissues if sufficient drug accumulation occurred outside tumors. The present study addresses this concern at several levels. BFMR-FA showed limited intrinsic cytotoxicity in normal cells, favorable hemocompatibility, stable serum biochemical and hematological parameters, and no obvious major-organ histological injury under the tested conditions. Moreover, the FA competitive uptake data suggest limited FA-mediated enrichment in nonmalignant liver cells. These findings support a therapeutic window based on tumor-preferential uptake, microenvironment-responsive activation, and the higher redox vulnerability of radioresistant tumor cells.

Several limitations should be acknowledged. Although this study provides pharmacological, biochemical, and oeGPX4-based functional evidence supporting ferroptosis involvement in radiosensitivity, the precise hierarchy among ferroptosis, apoptosis, and necroptosis-related pathways warrants further investigation. In addition, the immune functional analyses mainly focused on local tumor immune remodeling; future studies incorporating broader patient-derived models, long-term survival assessment, and systemic immune cell profiling will be valuable to further define the durability and translational relevance of BFMR-FA + RT.

**Fig. 9:**
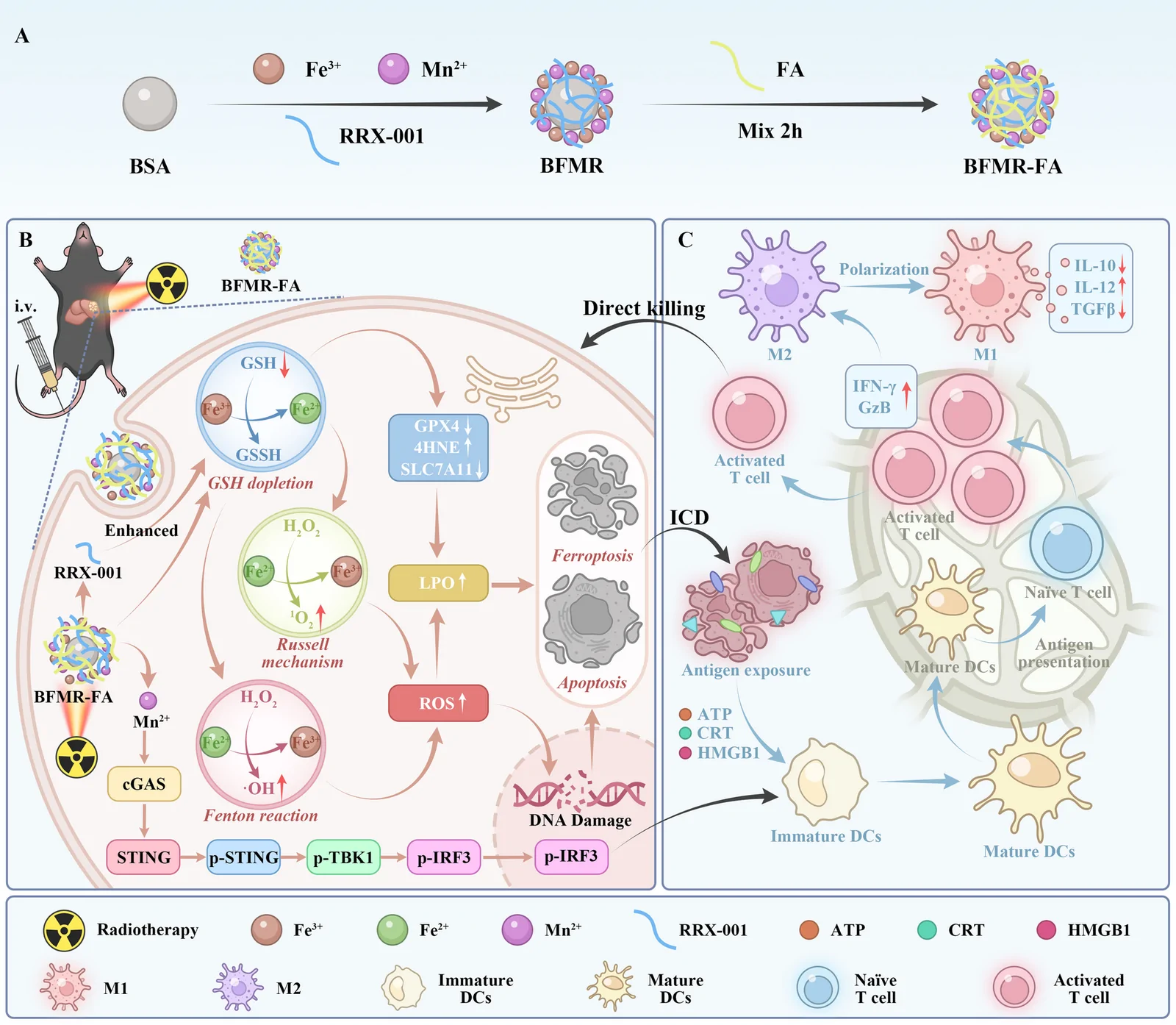
Schematic diagram displaying the overall process and mechanism of this study. A critical mechanistic fulcrum of this strategy is the disruption of the SLC7A11–GSH–GPX4 axis, a canonical redox axis universally elevated in therapy resistant tumors. RRX-001, incorporated into BFMR-FA, rapidly depletes reduced thiols via Michael addition, a mechanism with established clinical evidence. This redox disarming renders tumor cells acutely vulnerable to Fe/Mn-catalyzed hydroxyl radical generation and lipid peroxide amplification. Rather than merely increasing the ROS burden, BFMR-FA executes a bidirectional attack—depleting antioxidant defenses while intensifying oxidative stress—ultimately triggering a catastrophic collapse of oxidative homeostasis. This collapse provides a coherent explanation for why BFMR-FA achieves potent radiosensitization even at relatively low irradiation doses

In conclusion, BFMR-FA provides an image-guided redox-immune radiosensitization strategy for radioresistant HCC. By coupling tumor-preferential delivery with Fe/Mn-driven oxidative catalysis and RRX-001-mediated thiol disruption, BFMR-FA drives sustained lipid peroxidation and ferroptosis-centered mixed oxidative cell death under irradiation. This oxidative injury is accompanied by ICD-associated danger signaling, STING–TBK1–IRF3 activation, and local immune remodeling, thereby linking redox destabilization with antitumor immune activation. These coordinated effects convert radioresistant tumors from an oxidation-buffered and immune-suppressed state into a therapeutically vulnerable state, providing a mechanistic foundation for redox-immune integrated radiosensitization in HCC (Fig. 9).

## Supplementary Information

Supplementary Material 1.

Supplementary Material 2.

## Data Availability

The data used to support the findings of this study are available from the corresponding author upon request.
