# Supplementary material for: Coupling oxidative de-stabilization with immune activation overcomes radioresistance in hepatocellular carcinoma
Source: Mol Cancer. 2026 Jul 30;25:256. doi: 10.1186/s12943-026-02729-z (PMC13711812; doi:10.1186/s12943-026-02729-z)
Supplement: Supplementary file 1 [file 12943_2026_2729_MOESM1_ESM.docx]

**Coupling oxidative de-stabilization with immune activation overcomes radioresistance in hepatocellular carcinoma**

Jiahao Liu^a, 1^, Ze Mi^a, b, c, 1^, Hongpei Tan^a^, Junhong Duan^a^, Feilong Huang^a^, Shahan Guo^a^, Yuting Li^a^, Jianmin Wu^a^, Min Yang^a^, Yunjie Liao^a^, Xiaoqian Ma^a^, Qi Liang^a^, Weiwei Zhang^f, *^, Xiaofei Fan^d, e, *^, Pengfei Rong^a, g, *^

^a^ Department of Radiology, The Third Xiangya Hospital, Central South University, No. 138, Tongzipo Road, Changsha, Hunan, China, 410013

^b^ Department of Diagnostic Radiology, Yong Loo Lin School of Medicine, National University of Singapore, Singapore, 119074

^c^ Postdoctoral Station of Medical Aspects of Specific Environments, the Third Xiangya Hospital, Central South University, Changsha, China.

^d^ Shandong Medical College, No. 5460, Second Ring South Road, Jinan, Shandong, China, 250002

^e^ Transplantation Center, The Third Xiangya Hospital, Central South University, No. 138, Tongzipo Road, Changsha, Hunan, China, 410013

^f^ Department of Clinical Laboratory, Shandong Cancer Hospital and Institute, Shandong First Medical University, and Shandong Academy of Medical Sciences, Jinan, Shandong, China, 250117

^g^ Furong Laboratory, Changsha, Hunan, China 410008

[^1^] These authors contributed equally to this work.

[*] Corresponding author.

E-mail: [rongpengfei66@163.com](mailto:rongpengfei66@163.com) (P. Rong); fanxf_sdmc@163.com (X. Fan); wwzhang_vip@163.com (W. Zhang).

**Supporting Figures**

**Fig. S1.** TEM Images of FePt nanoparticles

**Fig. S2.** Effect of Fe:Mn molar ratio on the encapsulation efficiency (EE) and loading efficiency (LE) of RRX-001 in BFMR-FA nanoparticles. EE and LE were evaluated under different Fe:Mn ratios (5:1, 3:1, 1:1, 1:3 and 1:5).

**Fig. S3.** Flow cytometry analysis of intracellular reactive oxygen species (ROS) levels in cells treated with BFMR-FA nanoparticles synthesized at different Fe:Mn molar ratios. ROS generation was measured using a DCFH-DA fluorescent probe.

**Fig. S4.** Semi-quantitative quantification of (A) ROS levels and (B) the percentage of γ-H2AX–positive RR-Huh7 cells under different treatment conditions.

**Fig. S5.** CellROX staining validates BFMR-FA+RT-induced oxidative stress. **(A)** Representative CellROX fluorescence images of RR-HuH7 cells after different treatments, Scale bar = 100 μm. **(B)** Quantification of CellROX fluorescence intensity (n = 3).

**Fig. S6.** Semi-quantitative analysis of ROS levels (A, C) and γ-H2AX–positive rates (B, D) in radiosensitive and radioresistant HCC organoids (RR-HCC) following the indicated treatments.

**Fig. S7.** GPX4 overexpression mitigates BFMR-FA+RT-induced cytotoxicity. Cell viability of wt and oeGPX4 HCC cells after BFMR-FA+RT treatment. (A) HuH-7 cells. (B) Hepa1-6 cells (n = 5).

**Fig. S8.** Clonogenic assays of GPX4 overexpression (oeGPX4) on BFMR-FA+RT-induced tumor cell killing. Representative images and quantification of colony formation in HuH-7 (A, B) and Hepa1-6 (C, D) cells. Cells were treated with BFMR-FA+RT, and clonogenic survival was compared between control and oeGPX4 cells (n = 5).

**Fig. S9.** ELISA analysis of oxidative stress–related markers in different treatment groups. (A) GSH/GSSG ratio, (B) fold change of MDA and (C) fold change of GPX4 (n = 5).

**Fig. S10. Quantitative analysis of Western blot results in different treatment groups.** (A) 4-HNE/β-actin, (B) GPX4/β-actin, and (C) SLC7A11/β-actin (n = 3).

**Fig. S11. Quantitative analysis of immunofluorescence staining in different treatment groups:** (A) relative fluorescence intensity of SLC7A11, (B) relative fluorescence intensity of GPX4, and (C) relative fluorescence intensity of 4-HNE (n = 5).

**Fig. S12.** Seahorse analysis showing extracellular acidification rate (ECAR) (A) and oxygen consumption rate (OCR) (B) in control and BFMR-FA+RT–treated cells. Glucose, oligomycin, and 2-deoxyglucose (2-DG) were sequentially injected as indicated (n = 3).

**Fig. S13. BFMR-FA+RT promotes mitochondrial superoxide accumulation.(A)** Representative MitoSOX fluorescence images of RR-HuH7 cells after different treatments, Scale bar = 50 μm. **(B)** Quantification of MitoSOX fluorescence intensity (n = 3).

**Fig. S14. Quantitative analysis of C11-BODIPY staining in different treatment groups.** (A) relative fluorescence intensity of oxidized C11 and (B) relative fluorescence intensity of reduced C11 in RR-HuH7 cells.

.

**Fig. S15. Quantitative analysis of STING pathway-related Western blot results.** (A) p-STING/β-actin, (B) p-TBK1/β-actin, and (C) p-IRF3/β-actin in RR-HuH7 cells after different treatments.

**Fig. S16.** Semi-quantitative analysis of (A) HMGB1 release and (B) CRT exposure following the indicated treatments (n = 6).

**Fig. S17.** ELISA quantification of extracellular ATP (A) and HMGB1 (B) after the indicated treatments (n = 6).

**Fig. S18. Quantitative analysis of macrophage polarization-related Western blot results:** (A) CD86/β-actin and (B) CD206/β-actin in different treatment groups (n = 5).

**Fig. S19. FA-mediated cellular uptake of BFMR-FA in parental and radioresistant liver cancer cells.**
(A, B) CLSM images of Cy5.5-labeled BFMR and BFMR-FA uptake in Hu-H7 and RR-Hu-H7 cells with or without excess free FA pretreatment. Blue fluorescence indicates DAPI-stained nuclei, and red fluorescence indicates Cy5.5-labeled nanoparticles. (C) Quantitative analysis of relative Cy5.5 fluorescence intensity in Hu-H7 and RR-Hu-H7 cells (n = 5, Scale bar = 50 μm). (D, E) CLSM images showing cellular uptake of Cy5.5-labeled BFMR and BFMR-FA in Hepa1-6 and RR-Hepa1-6 cells with or without excess free FA pretreatment. (F) Quantitative analysis of relative Cy5.5 fluorescence intensity in Hepa1-6 and RR-Hepa1-6 cells (n = 5, Scale bar = 50 μm). (G, H) CLSM images showing cellular uptake of Cy5.5-labeled BFMR and BFMR-FA in LO2 and AML-12 cells. (I) Quantitative analysis of relative Cy5.5 fluorescence intensity in LO2 and AML-12 cells (n = 5, Scale bar = 50 μm).

**Fig. S20.** Representative fluorescence images of tumor sections from mice treated with Cy5.5-labeled (A) BFMR or (B) BFMR-FA. The upper panels show whole-tumor fluorescence distribution (scale bar = 5 mm), while the lower panels present higher-magnification images highlighting intracellular localization of Cy5.5 signals within tumor cells (scale bar = 20 μm). Cy5.5 fluorescence (red) indicates nanoparticle accumulation and DAPI (blue) labels cell nuclei.

**Fig. S21. Quantitative analysis of apoptosis-related immunofluorescence staining in tumor tissues.** (A) relative fluorescence intensity of TUNEL and (B) relative fluorescence intensity of cleaved caspase-3 in different treatment groups (n = 3).

**Fig. S22. Quantitative analysis of immunofluorescence staining in tumor tissues.**
Relative fluorescence intensity of tumor immune markers in different treatment groups: **(A)** CD11B, **(B)** CD86, and **(C)** CD206 (n = 5).
