# Supplementary material for: Coupling oxidative de-stabilization with immune activation overcomes radioresistance in hepatocellular carcinoma
Source: Mol Cancer. 2026 Jul 30;25:256. doi: 10.1186/s12943-026-02729-z (PMC13711812; doi:10.1186/s12943-026-02729-z)
Supplement: Supplementary file 2 [file 12943_2026_2729_MOESM2_ESM.docx]

4-HNE and actin of Fig. 3B

GPX4 and actin of Fig. 3E

SLC7A11 and actin Fig. 4B

Sting and actin of Fig. 4B

p-Sting and actin of Fig. 4B

TBK1 and actin of Fig. 4B

p-TBK1 and actin of Fig. 4B

IRF3 and actin of Fig. 4B

p-IRF3 and actin of Fig. 4B

CD86 and actin of Fig. 4I

CD206 and actin of Fig. 4I
